# A Computational Model of a Single Auditory Nerve Fiber for Electric-Acoustic Stimulation

**DOI:** 10.1007/s10162-022-00870-2

**Published:** 2022-11-04

**Authors:** Daniel Kipping, Waldo Nogueira

**Affiliations:** 1 Department of Otolaryngology, Hannover Medical School (MHH), Hannover, Germany; 2 Cluster of Excellence Hearing4all, Hannover, Germany

**Keywords:** Cochlear implants, Auditory nerve fibers, Computational models, Electric-acoustic stimulation, Electric-acoustic interaction, Spike timing statistics

## Abstract

Cochlear implant (CI) recipients with preserved acoustic low-frequency hearing in the implanted ear are a growing group among traditional CI users who benefit from hybrid electric-acoustic stimulation (EAS). However, combined ipsilateral electric and acoustic stimulation also introduces interactions between the two modalities that can affect the performance of EAS users. A computational model of a single auditory nerve fiber that is excited by EAS was developed to study the interaction between electric and acoustic stimulation. Two existing models of sole electric or acoustic stimulation were coupled to simulate responses to combined EAS. Different methods of combining both models were implemented. In the coupled model variant, the refractoriness of the simulated fiber leads to suppressive interaction between electrically evoked and acoustically evoked spikes as well as spontaneous activity. The second model variant is an uncoupled EAS model without electric-acoustic interaction. By comparing predictions between the coupled and the noninteracting EAS model, it was possible to infer electric-acoustic interaction at the level of the auditory nerve. The EAS model was used to simulate fiber populations with realistic inter-unit variability, where each unit was represented by the single-fiber model. Predicted thresholds and dynamic ranges, spike rates, latencies, jitter, and vector strengths were compared to empirical data. The presented EAS model provides a framework for future studies of peripheral electric-acoustic interaction.

**Supplementary Information:**

The online version contains supplementary material available at 10.1007/s10162-022-00870-2.

## Introduction

Due to the relaxation of cochlear implant (CI) candidacy criteria, patients with asymmetric severe to profound hearing loss in the high frequencies but good residual low-frequency hearing may also be eligible for a CI [1–4]. Atraumatic surgical techniques and electrode designs can be used to preserve their acoustic hearing [5, 6]. CI users with a residual hearing in the implanted ear benefit greatly from the synergies between combined electric-acoustic stimulation (EAS), which offers improved speech perception when compared to traditional CI users that rely completely on electric input [7–10].

Nonetheless, hybrid EAS can also cause interactions between both stimulation modalities that can hamper the perception of electric stimulation (ES) and acoustic stimulation (AS) and even limit the benefits in speech intelligibility [11]. Signals presented with ES and AS can mask each other when presented simultaneously [12–15] or in succession [16]. The origin of EAS interaction seems to be at least partly in the periphery as several studies using electrocochleography and electrically evoked compound action potentials in EAS users have shown [17–20]. This observation is consistent with animal studies, which showed that EAS interaction already affects the activity in single auditory nerve fibers (ANFs) [21, 22] as well as electrically and acoustically evoked compound action potentials [23–26]. In animals, ES and AS can also interact through electrophonic stimulation of hair cells [27–32]. In human EAS users, however, electrophony seems to be less relevant due to high-frequency hearing loss [33, 34] and was shown not to contribute to psychoacoustic EAS masking [12]. Therefore, it can be assumed that peripheral EAS interaction, relevant to human EAS subjects, originates in the auditory nerve. Nevertheless, it is an open question how exactly the behavioral or electrophysiological EAS interaction observed in humans relates to the fundamental interactions observed in animal studies.

Computational models are widely used to simulate the activity in the auditory nerve in response to AS [35–37] or ES [38–43]. Apart from studying neural coding itself, models of single-ANF activity have been used as a front end for more complex models, for instance, to simulate electrophysiological measures [44–47] or the activity at central auditory nuclei [37, 48–51]. However, such modeling has been restricted to either AS of hearing ears or ES of deaf ears. In order to study the effects of peripheral EAS interaction in silico, an efficient model of single-ANF activity in response to hybrid EAS is necessary.

This paper presents a novel single fiber model of combined EAS that is based on two phenomenological models of either electroneurally [42] or acoustically [35] driven feline single-ANF activity. The EAS model can simulate ANF spike times for different degrees of hearing loss, ranging from completely deaf to normal hearing, and reproduces realistic spiking in response to ES, AS, and combined EAS. By varying several model parameters, modeled populations of ANFs can mimic realistic interunit variability. We investigate different coupling variants between the two models in order to assess EAS interaction effects that are caused by the refractoriness of the ANF. Interaction through electrophonic excitation of hair cells is not simulated by the model, although it has been observed in animals with preserved acoustic hearing [28, 33, 52, 53]. However, in human EAS users, their severe high-frequency hearing loss makes electrophonic stimulation unlikely with current clinical stimulation strategies that use short pulses [12, 33, 34]. Thus, as in the presented model, ANF responses and peripheral EAS interactions in human subjects are presumably generated by direct electroneural stimulation of ANFs together with AS. When combined with an existing simulation framework of clinically relevant measures, the model has the potential to assess EAS interaction along the auditory pathway and to relate the EAS interaction observed in human EAS subjects to underlying fundamental interactions at the level of the AN.

## Methods and Materials

The novel EAS model is comprised of two main building blocks that simulate feline ANF responses for either ES or AS. The first block, the ES model, simulates the direct electric ANF stimulation through an implanted electrode. It consists of an extended version of the adaptive integrate-and-fire model of Joshi et al. [42]. The second block is the AS model, which simulates the acoustically evoked ANF activity and consists of the phenomenological model of Bruce et al. [35]. Two different variants of the EAS model are presented to simulate an ANF either with or without interaction between the ES model and the AS model. The following sections introduce the ES model, the AS model, and the coupling between both models. Subsequently, the simulations performed for the results section are explained.

The code for the presented EAS model is available at Zenodo (https://doi.org/10.5281/zenodo.5467990) and GitHub (https://github.com/APGDHZ/Single-fiber-EAS-model/tree/v1.0.0).

### ES Model

#### Model Structure

The ES model is based on the adaptive integrate-and-fire model developed by Joshi et al. [42], which simulates the activity of a single ANF in response to electroneural stimulation. The model has been fitted to experimental data derived from deaf cats and does not include input from an IHC-ANF synapse. The self-implemented version incorporated in the EAS model extends the model of Joshi et al. [42] by a new randomized parameter set that accounts for interfiber variability in thresholds, relative spread (RS), and refractoriness in ANF populations.

**Fig. 1:**
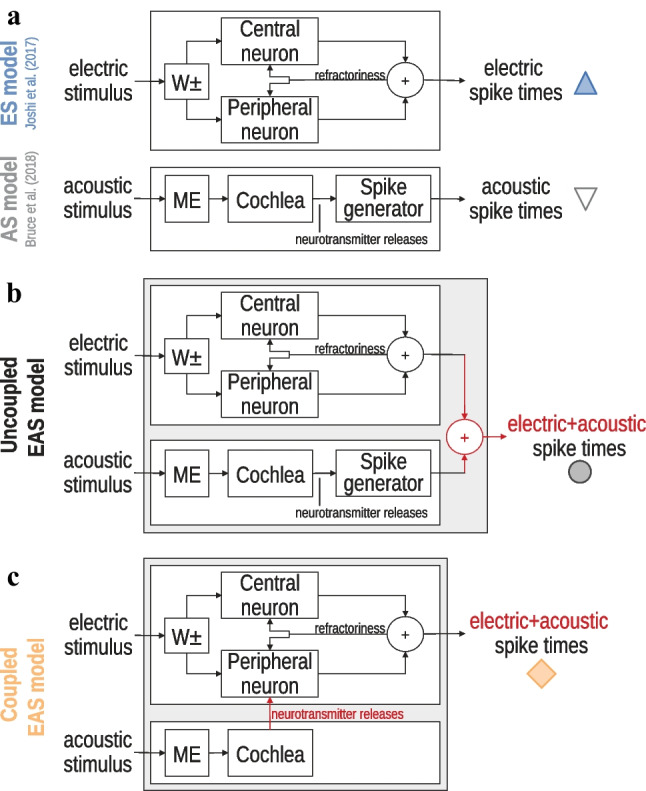
Block diagrams for the used models. The input to each model is the waveform of an electric or acoustic stimulus or a combination of both and the output is an array of spike times in the ANF. The symbols indicated next to each model output are used in the results section to represent the respective model results. **a** Top: extended ES model based on Joshi et al. [42], bottom: AS model of Bruce et al. [35]. **b** Uncoupled EAS model without interaction. **c** Coupled EAS model: the neurotransmitter release events from the acoustic IHC model trigger an additional excitatory input current to the peripheral neuron of the ES model, bypassing the acoustic spike generator. W ± – stimulus weighting block, Central/peripheral neuron—adaptive integrate-and-fire point neuron models with sub- and suprathreshold feedback currents; ME—middle ear filter; Cochlea—includes basilar membrane, outer hair cells, inner hair cells, and the synapse; Spike generator—generates spikes by accounting for the refractoriness of the ANF

In the ES model, spikes can be generated either in the peripheral or the central process of the ANF. The processes are represented by two structurally identical integrate-and-fire point neuron models, which account for the different excitatory and inhibitory characteristics (Fig. 1a, top). Throughout this paper, inhibition of a neuron refers to direct electrical suppression of the neuron due to an extracellular current injection. Cathodic current excites the peripheral neuron and inhibits the central neuron, whereas anodic current excites the central neuron and inhibits the peripheral neuron. The cathodic (${I}^{-}$) and anodic (${I}^{+}$) contributions to the stimulating currents for the two point neurons are weighted according to

1a $${I}_{stim}^{peripheral}\left(t\right)=-\left({I}^{-}\left(t\right)+\beta {I}^{+}\left(t\right)\right)\,,$$

1b $${I}_{stim}^{central}\left(t\right)=\beta {I}^{-}\left(t\right)+{I}^{+}\left(t\right)\,,$$

where $\beta$ quantifies the amount of the inhibitory charge that enters the neuronal membranes (weighting block “W ± ” in Fig. 1a).

The time course of the cross-membrane voltages $V$ of the neurons is given by

2 $$C\frac{dV}{dt}=h\left(V\right)-{I}_{sub}-{I}_{supra}+{I}_{noise}+{I}_{stim}\,,$$

where $C$ is the membrane capacitance, ${I}_{sub}$ and ${I}_{supra}$ are sub- and suprathreshold adaptation currents, and ${I}_{stim}$ is the stimulating current defined in Eq. (1). ${I}_{noise}$ is a noise source with variance ${\sigma }_{noise}^{2}$ and a frequency spectrum with power spectral density of $1/{f}^{\alpha}$. The function $h(V)$ provides the passive filtering of the membrane as well as the spiking mechanism:

3 $$h\left(V\right)=-{g}_{L}\left(V-{E}_{L}\right)+{g}_{L}{\Delta }_{T}{e}^{\left(V-{V}_{T}\right)/{\Delta}_{T}}\,,$$

where ${g}_{L}$ is the membrane conductance, ${E}_{L}$ is the reversal potential, ${\Delta}_{T}$ is the slope factor for the exponential upswing of the membrane voltage during spiking, and ${V}_{T}$ is the threshold potential.

The subthreshold and suprathreshold adaptation currents evolve according to

4 $${\tau }_{sub}\frac{d{I}_{sub}}{dt}={a}_{sub}\left(V-{E}_{L}\right)-{I}_{sub}\,,$$

5 $${\tau }_{supra}\frac{d{I}_{supra}}{dt}={a}_{supra}\left(V-{E}_{L}\right)-{I}_{supra}\,,$$

where ${\tau}_{sub}$ and ${\tau}_{supra}$ are sub- and suprathreshold adaptation time constants and ${a}_{sub}$ and ${a}_{supra}$ are sub- and suprathreshold adaptation conductances, respectively. Equations (2)–(5) are applied to both point neuron models, with different parameter sets for the peripheral and the central process of the ANF.

In the absence of spikes, the membrane voltages of the peripheral and the central neuron are integrated independently from each other. A spike in the ANF is indicated when the membrane voltage of one of the neurons crosses the spike detection threshold ${V}_{spike}$. Following spiking, a post-spike adaptation process is applied to both neurons irrespective of which neuron produced the action potential: (i) the membrane voltages are reset to ${V}_{reset}$; (ii) the suprathreshold adaptation currents are increased by a constant $b$ to produce a hyperdepolarization of the neurons; and (iii) the neurons are set into an absolute refractory state with a duration ${t}_{dead}$.

6 $$V\to {v}_{res}\text{ and }{I}_{supra}\to {I}_{supra}+b \;\text{at the time of spiking}$$

Refractoriness consists of two parts, the absolute refractory period (ARP) followed by the relative refractory period (RRP). During the ARP, it is impossible to generate another spike irrespective of the stimulus amplitude. Therefore, in the ES model, the neurons do not receive input from any further stimulation during the ARP. The RRP describes the subsequent relaxation of the elevated spike threshold back to its resting value and is realized through the suprathreshold adaptation currents.

#### Extension to an ES Population Model

In its original form published by Joshi et al. [42], the model parameters have been fitted to reproduce responses of an “average” ANF based on several experimental studies. Therefore, the original ES model could not account for the variability between different ANFs. For the new EAS model, we modified the ES model proposed by Joshi et al. [42] and randomized the membrane capacitances $C$ to obtain realistic distributions of ANF thresholds (Appendix 1).

Moreover, in order to produce consistent refractory durations after electrically or acoustically evoked spiking in the same ANF, the ARP (${t}_{dead}$) and the RRP (time constant ${\tau}_{supra}$) of the ES model were modified according to the randomized refractory time constants of the AS model (${t}_{abs}$ and ${t}_{rel}$):

7 $${t}_{dead}^{(el)}={t}_{abs}^{(ac)}\,,$$

8 $${\tau}_{supra}^{(el)}={\tau }_{supra,0}\cdot \frac{{t}_{rel}^{(ac)}}{\langle {t}_{rel}^{(ac)}\rangle } \,,$$

where ${\tau }_{supra,0}$ is the original value of the suprathreshold adaptation time constant from the Joshi et al. [42] model, and ${t}_{rel}^{(ac)}/\langle {t}_{rel}^{(ac)}\rangle$ is the baseline RRP of the AS model normalized to its expectation value (see Table 2). Where necessary, the parameters of the ES model and the AS model are marked with the superscripts “*ac*” or “*el*” to distinguish them from each other. While the parameters ${t}_{dead}$ and ${\tau}_{supra}$ have been fixed in the original publication of the ES model, they are now randomized according to the distributions of ${t}_{abs}$ and ${t}_{rel}$ in the AS model. In consequence, the mean dead time of the ES model has been slightly decreased from 500 to 450 $\mu \mathrm{s}$.

In addition to the parameter changes described above, also the implementation of the ES model differed slightly from the original implementation described by Joshi et al. [42]. In the study of Joshi et al. [42] the membrane was always in a deterministic initial state $V={E}_{L}$, ${I}_{sub}={I}_{supra}=0$ when the stimulus was applied. The implementation used for the present study considers more realistically that in absence of stimulation the membrane is in a random state produced by evolving the neuron in time with the random noise current ${I}_{noise}$. This new implementation produced larger jitter and RS because the model responded less deterministically. Therefore, the noise amplitudes ${\sigma }_{noise}$ were refitted to best reproduce the RS to monophasic stimulation, and the slope factor $\Delta_T$ for the central neuron was reduced from 4.0 to 3.0 mV to produce lower jitter in response to monophasic anodic stimulation. Smaller slope factors produce a faster upswing of the exponential term in Eq. (3) during spiking and thereby reduce spike latency and jitter.

The parameters used for the ES population model are summarized in Table 1. Changes in comparison to the original model version by Joshi et al. are indicated with symbols (*, †).

**Table 1:** Parameter set for the ES population model. Differences to the original model by Joshi et al. [42] are indicated with asterisks (*). Parameters linked to parameters of the AS model (superscript “ac”) are marked with crosses (†)

	**Peripheral neuron**	**Central neuron**
**Membrane conductance** ${\varvec{g}}_{\varvec{L}}$	**1.1 mS**	**2.7 mS**
Membrane capacitance $C$ (*) $C={10}^{x}-\alpha$ with $x\sim \mathcal{N}(\mu ,{\sigma }^{2})$ normally distributed and truncated at $\mu \pm 2\sigma$ See Appendix 1	$\mu =-6.1514$ $\sigma =0.1947$ $\alpha =-164.0\,\mathrm{nF}$	$\mu =-5.7547$ $\sigma =0.2010$ $\alpha =-32.7\,\mathrm{nF}$
Slope factor $\Delta_T$ (*)	10.0 mV	3.0 mV
Resting potential ${E}_{L}$	− 80.0 mV	
Threshold potential ${v}_{thr}$	− 70.0 mV	
Peak potential ${v}_{peak}$	24.0 mV	
Reset potential ${v}_{reset}$	− 84.0 mV	
Noise shaping parameter $\alpha$	0.80	
Inhibitory compression $\beta$	0.75	
Subthreshold adaptation time constant ${\tau}_{sub}$	250.0 µs	
Suprathreshold adaptation time constant ${\tau }_{supra}$ (†)	$4500.0\,\mu \mathrm{s}\cdot \frac{{t}_{rel}^{(ac)}}{\langle {t}_{rel}^{(ac)}\rangle }$	$2500.0\,\mu \mathrm{s}\cdot \frac{{t}_{rel}^{(ac)}}{\langle {t}_{rel}^{(ac)}\rangle }$
Subthreshold adaptation conductance ${a}_{sub}$	2.0 mS	
Suprathreshold adaptation conductance ${a}_{supra}$	3.0 mS	
Dead time ${t}_{dead}$ (†)	${t}_{abs}^{(ac)}$	
Noise amplitude ${\sigma}_{noise}$ (*)	8.70 µA	11.89 µA
Spike-triggered offset for suprathreshold adaptation currents $b$	90.0 µA	

### AS Model

The acoustic hearing pathway including the middle ear (ME), basilar membrane (BM), outer and inner hair cells (OHCs and IHCs), and the IHC-AN synapse is simulated by a phenomenological model of the auditory periphery [35]. Figure 1a shows the structure of this AS model. The input to the model is an acoustic sound-pressure wave, and the output is simulated spike times in a single ANF at a time resolution of 10 µs. The input sound-pressure wave is processed by a ME filter and then passed to a “cochlea” block. The “cochlea” block includes filters for the tuning of the BM to the CF of the fiber, a control path mimicking the fine-tuning of the BM by the electromotility of the OHCs, an IHC module, which simulates the resulting IHC cross-membrane potential, and a synapse model that simulates the events of neurotransmitter releases into the IHC-ANF synaptic cleft. This synapse model implements exponentially distributed redocking and release times to account for the stochasticity of the neurotransmitter release events. The final step is a spike generator, which converts the neurotransmitter release times into spike times at the ANF and implements the refractoriness of the ANF based on ${t}_{abs}$ and ${t}_{rel}$.

The ANF is characterized by a parameter set consisting of the characteristic frequency (CF), the spontaneous spike rate (SR), parameters for the impairment of OHCs and IHCs ($${c}_{OHC}$$ and $${c}_{IHC}$$), and the mean durations of the ARP ($${t}_{abs})$$ and baseline RRP ($${t}_{rel}$$).

### EAS Coupling

The two models introduced in the previous sections simulate the spike timing

- of a fiber of a deaf ear that is stimulated electrically but without input from an IHC-ANF synapse (ES model); or
- of an acoustically sensitive fiber (including spontaneous spiking) but in the absence of ES (AS model).

To distinguish them from the novel EAS model, we will sometimes refer to these baseline models as the ES alone model and the AS alone model.

First of all, to produce consistent refractory behavior for ES and AS of the same ANF, the parameters affecting the refractoriness of the fiber have been correlated between the ES alone model and the AS model as described above.

Secondly, to simulate spike timing in an acoustically sensitive ANF, which is excited by combined EAS, a coupled EAS model has been designed. This means that both models run simultaneously and exchange information to implement the interaction between acoustic and electric excitations in the same ANF. The different EAS model variants presented in the following paragraphs are also represented in Fig. 1.

#### Uncoupled EAS Model (Fig. 1b)

The first variant of the EAS model is a simple baseline model where the spike times generated by both models are merged. The uncoupled EAS model does not include any interaction between the ES model and the AS model. This model can be used as a reference to assess the influence of EAS interaction contained in the coupled EAS model.

#### Coupled EAS Model (Fig. 1c)

Inspired by the physiological function of the IHC-ANF synapse, this interacting EAS model mimics the generation of postsynaptic action potentials in the peripheral ANF axon that are triggered by neurotransmitter released from the IHC ribbon into the synaptic cleft. The AS model simulates the neurotransmitter release events with a synapse model (“cochlea” block in Fig. 1). In the original AS model by Bruce et al. [35], the “spike generator” then generated an action potential for every neurotransmitter release event, unless the ANF was in the refractory state. Neurotransmitter releases occurring during refractoriness did not result in spikes nor did they contribute to any further excitation of the ANF.

In the coupled EAS model, the spike generator of the AS model is replaced by the peripheral axon of the ES model. Each neurotransmitter release event is converted into an additional excitatory (i.e., cathodic) input current ${I}_{neuro}$ entering the neuronal membrane of the peripheral neuron of the ES model (Eq. (2)). In order to preserve the spiking characteristics of the original AS model, every injected synaptic current must lead to an action potential in the peripheral integrate-and-fire neuron, unless this neuron is in refractory state. Thus, the synaptic current needs to be substantially suprathreshold. In this suprathreshold regime, the exact waveform of ${I}_{neuro}$ is not essential. For simplicity, the coupled EAS model uses a rectangular current injection with a duration ${t}_{neuro}=40\; \mu \mathrm{s}$ and an amplitude ${I}_{neuro}=3.0 \;\mathrm{mA}$ identical to a monophasic pulse. The amplitude is high enough to elicit an action potential in the peripheral neuron irrespective of the individual ANF parameters unless the ES model is in the refractory state and thus replicates the behavior of the original spike generator of the AS model.

### Experiments

Three experiments were conducted. All simulations were performed for a population of 150 ANFs (30 LSR, 30 MSR, 90 HSR) with parameters drawn according to Table 2. In the following, the term “acoustically insensitive” is used for ANFs simulated with the ES alone model, and “acoustically sensitive” is used for ANFs simulated with the EAS model variants (uncoupled or coupled). This implies the simplification that only “acoustically sensitive” ANFs show spontaneous activity.

**Table 2:** Parameter distributions of the EAS population model

**Parameter**	**Value**	**Comment/Reference**
Characteristic frequency ${\mathrm{CF}}^{(ac)}$ in $[\mathrm{kHz}]$	Logarithmic distribution from 0.125 to 40.0	Liberman and Kiang [54]
Spontaneous spike rate ${\mathrm{SR}}^{(ac)}$ in $[\mathrm{spikes}/\mathrm{s}]$	Gaussian distribution LSR: $\mu =0.1$, $\sigma =0.1$, limits $[{10}^{-3} , 0.2]$ MSR: $\mu =4.0$, $\sigma =4.0$, limits $[0.2 , 18.0]$ HSR: $\mu =70.0$, $\sigma =30.0$, limits $[18.0 , 180.0]$	Bruce et al. [35]
ARP ${t}_{abs}^{(ac)}$ in $[\mu \mathrm{s}]$	Uniform dist.: limits $[208.5 , 691.5]$	Bruce et al. [35]
Baseline RRP ${t}_{rel}^{(ac)}$ in $[\mu \mathrm{s}]$	Uniform dist.: limits $[131.0 , 894.0]$	Bruce et al. [35]
Membrane capacitances ${C}^{(el)}$	See Table 1	Fitted to single fiber thresholds from Miller et al. [55], see Appendix 1
Suprathreshold adaptation time constants ${\tau}_{supra}^{(el)}$	See Table 1	To get similar refractory behavior of the ES and AS models
Dead time: ${t}_{dead}^{(el)}$	${{t}_{dead}^{(el)}=t}_{abs}^{(ac)}$	To get similar refractory behavior of the ES and AS models

#### Experiment 1: The Extended ES Alone Model

Experiment 1 investigated electric-only stimulation of acoustically insensitive ANFs (see section “ES Model”). The main purpose was to validate the new implementation and extension of the ES model. The ES model was fitted based on the responses to monophasic single pulses. It has subsequently been validated for symmetric biphasic pulses and paired-pulse paradigms with either sub- or suprathreshold conditioners. The fitting and validation for electric-only stimulation generally followed the experimental settings of Joshi et al. [42], using the data of Dynes [56] and Miller et al. [55, 57].

#### Experiment 2: The Novel EAS Model with Electric-Only Stimulation

Experiment 2 investigated electric-only stimulation of acoustically sensitive ANFs as in the study of Miller et al. [58]. ES consisted of pulse trains of symmetric biphasic pulses presented at 250 pulses per second (pps). The focus was on the influence of spontaneous spiking generated by the AS model on the electrically evoked responses. Simulations were performed with the uncoupled and the coupled EAS model as well as the ES and AS alone models. The impairment of IHCs and OHCs (${c}_{IHC}$ and ${c}_{OHC}$) was determined according to the average hearing loss of 26 dB HL reported by Miller et al. [21, 58]. The influence of EAS interaction was assessed by comparing the predictions of the coupled EAS model with the outcomes of the uncoupled EAS model and the ES alone model.

#### Experiment 3: The Novel EAS Model with Electric-Acoustic Stimulation

Experiment 3 investigated combined EAS of acoustically sensitive ANFs using the uncoupled and the coupled EAS model. The experimental paradigm was the same as in the study of Miller et al. [21]. Pulse trains identical to experiment 2 were used for ES, and data was obtained at four different current levels per ANF randomly selected across the DR of the fiber. AS consisted of acoustic broadband noise at levels between 70 and 100 dB SPL. As in experiment 2, a flat hearing loss of 26 dB HL was assumed to determine the status of IHCs and OHCs.

### Response Statistics

For single-pulse ES, the firing efficiency (FE) at a certain stimulus level was computed by simulating the responses to 100 repetitions of the same stimulus. FE was defined as

9 $$\mathrm{FE}=(N-\mathrm{SR}\cdot T)/M\;,$$

where $N$ was the number of action potentials observed in a specified analysis window with a duration $T$, $\mathrm{SR}$ was the spontaneous rate of the ANF, and $M$ was the number of stimulus presentations [53]. For ES with pulse trains, $M$ was the total number of pulses in all repeated stimulus presentations, and at least 15 pulse train presentations were used. When reporting electrically or acoustically driven spike rates for experiment 3, the raw spike rates were always corrected for the SR of the ANF. Threshold and relative spread (RS) were estimated by fitting an integrated Gaussian to the FE-level data. The threshold was defined as the stimulus level which evoked a FE of 50 % and corresponds to the mean $\mu$ of the underlying Gaussian distribution. The RS is the ratio $\mathrm{RS}=\sigma /\mu$ of the standard deviation to the mean and can be used as a measure of the fiber’s electric dynamic range (DR).

Spike timing was assessed through mean spike latencies (relative to the pulse onset) and jitter, defined as the standard deviation of the spike latencies. For ES with pulse trains, the synchrony of spike timing relative to the electric pulses was measured in terms of the vector strength (VS) [59]

10 $$\mathrm{VS}=\frac{1}{N}\sqrt{{\left({\sum }_{\mathrm{i}}\mathrm{sin}(2\pi \nu {t}_{i})\right)}^{2}+{\left({\sum }_{\mathrm{i}}\mathrm{cos}(2\pi \nu {t}_{i})\right)}^{2}} \ ,$$

where ${t}_{i}$ are the spike times, $N$ is the total number of spikes, and $\nu$ is the pulse rate of the stimulus.

## Results

### Experiment 1: The Extended ES Alone Model

Experiment 1 investigated the ES of acoustically insensitive ANFs represented by the ES alone model without the additional AS model component.

**Fig. 2:**
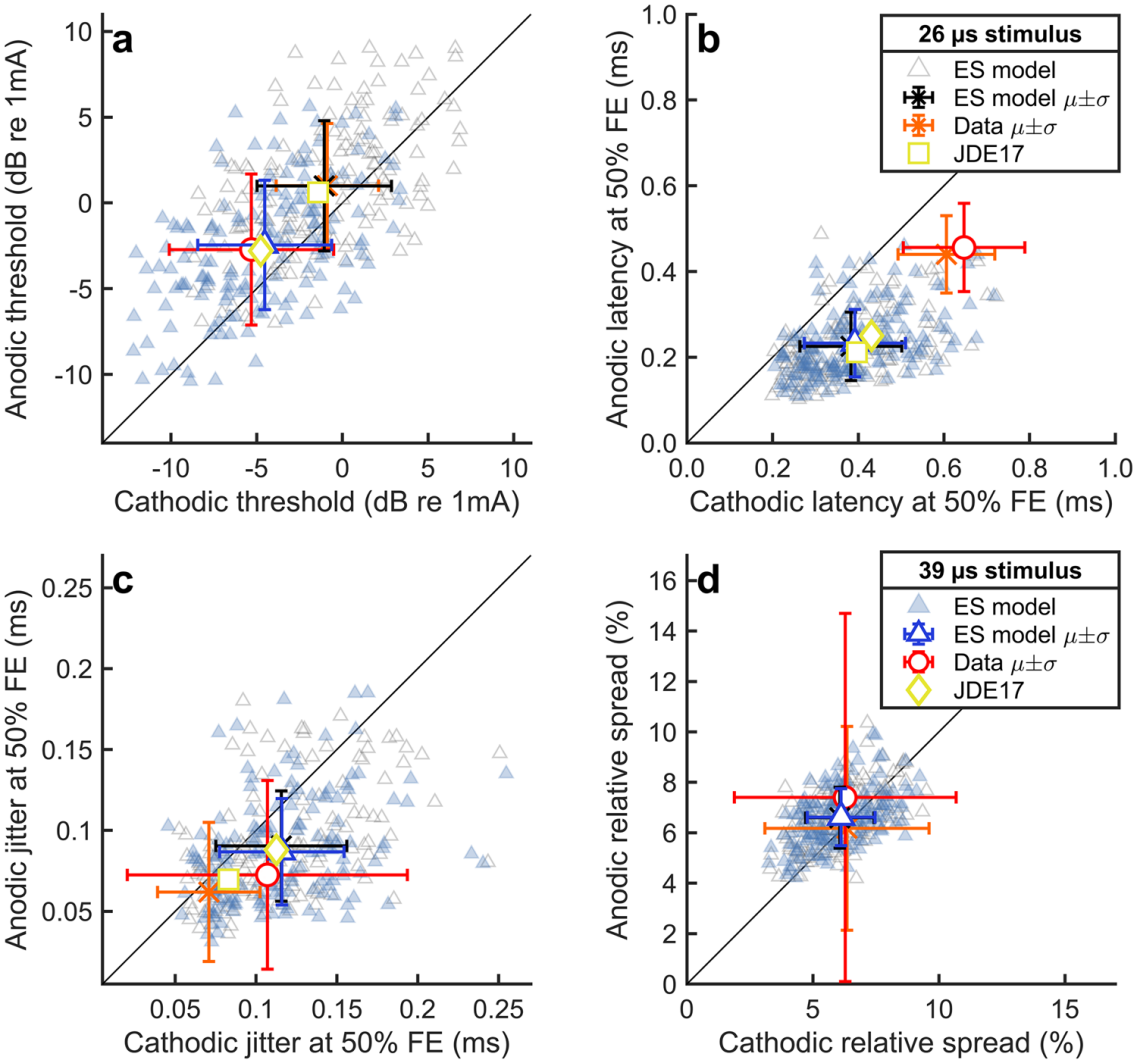
Response statistics of the extended ES alone model for monophasic anodic and cathodic pulses with a duration of 26 µs (grey triangles) and 39 µs (blue triangles). **a** Thresholds, defined as the current resulting in a FE of 50 %. **b**, **c** Latency and jitter when stimulating at threshold level. **d** Relative spread. Mean values (µ) are compared to the corresponding mean experimental data from Miller et al. [55] (see figure legend) and predictions of the original ES model of Joshi et al. [42] (yellow symbols, “JDE17”). Error bars represent the standard deviation (σ) of the data

Figure 2 presents thresholds (a), mean spike latencies (b), jitter (c), and RS (d) for stimulation with single monophasic current pulses (26 µs or 39 µs phase duration). Each subplot shows the predicted response statistics along with the mean responses of an experimental ANF population [55]. Error bars depict the standard deviations of the experimental or simulated data sets. For comparison, the available predictions of the original ES model [42] were included in panels a–c. The threshold distributions (a) have been used to derive the membrane capacitance distributions, and the noise current amplitudes were fitted to the mean RS (d) (see Appendix 1). Latency (b) and jitter (c) were a result of these parameter settings. Both latency and jitter were mainly determined by the membrane capacitance and largely independent of the noise amplitudes. Figure 2 shows that cathodic stimulation resulted in lower thresholds, but higher latency and jitter than anodic stimulation for both the model and the experimental data, whereas RS was similar for both stimulation polarities.

Table 3 compares the mean values and variances of the data shown in Fig. 2 quantitatively. The mean threshold and RS were consistent between model predictions and data, as well as the jitter for 39 µs stimuli. However, the jitter for short pulses of 26 µs was overestimated by the ES model. The reason may have been that in the new implementation of the ES model, the jitter for short pulses was increased due to the nondeterministic initial state of the membrane. The original model [42] yielded more accurate predictions for jitter by assuming a deterministic membrane state at the stimulus onset. The mean spike latencies predicted by the ES model were lower than observed in the experiment, a result in line with the original model version by Joshi et al. [42], which also predicted latencies lowered by about 200 µs when compared to the data of Miller et al. [55] (Fig. 2b). Overall, the variance across ANFs predicted by the extended ES model was reasonable for thresholds, latency, and jitter. Only for cathodic thresholds (26 µs stimulus) and cathodic and anodic jitter (39 µs stimulus), the difference between predicted and experimental standard deviation was larger than 30 %. However, the predicted variance in RS was generally low when compared to the empirical data. This point is addressed later in the discussion.

**Table 3:** Predictions of the ES model and corresponding data from Miller et al. [47, 55] for monophasic stimulation. The values represent the mean ± standard deviation across the ANF population

			**26 µs stimulus**		**39 µs stimulus**	
			**Cathodic**	**Anodic**	**Cathodic**	**Anodic**
Threshold (dB re 1 mA)		Model	− 1.06 ± 3.92	1.00 ± 3.80	− 4.53 ± 3.91	− 2.46 ± 3.77
		Data	− 0.88 ± 2.99	1.00 ± 3.64	− 5.31 ± 4.80	− 2.72 ± 4.40
Mean latency (µs)		Model	383 ± 119	225 ± 80	392 ± 118	233 ± 79
		Data	606 ± 113	440 ± 90	647 ± 142	456 ± 103
Jitter (µs)		Model	115.6 ± 40.6	90.3 ± 34.1	115.9 ± 38.5	86.8 ± 32.9
		Data	70.8 ± 31.7	61.9 ± 43.0	107.0 ± 86.6	72.5 ± 58.3
Relative spread (%)		Model	6.07 ± 1.36	6.60 ± 1.21	6.12 ± 1.34	6.62 ± 1.13
		Data	6.35 ± 3.26	6.18 ± 4.04	6.28 ± 4.40	7.40 ± 7.30

Additional simulation results validating the ES population model for biphasic stimulation and sub- and suprathreshold maskers are presented in Appendix 2.

### Experiment 2: The Novel EAS Model with Electric-Only Stimulation

Experiment 2 simulated the responses of acoustically sensitive ANFs to ES. Results obtained with the uncoupled and the coupled EAS population model were compared to the study of Miller et al. [58]. The electric stimulus was a constant-amplitude pulse train with a duration of 300 ms and consisted of 40 µs/phase biphasic (cathodic leading) pulses presented at 250 pps. Although no AS was presented, the spontaneous activity generated by the IHC in the AS model potentially affected the response behavior of the ANFs to ES. The responses predicted for acoustically sensitive ANFs with EAS interaction (coupled EAS model) were compared to responses of acoustically insensitive ANFs (ES alone model) and responses of ANFs without EAS interaction (uncoupled EAS model).

**Fig. 3:**
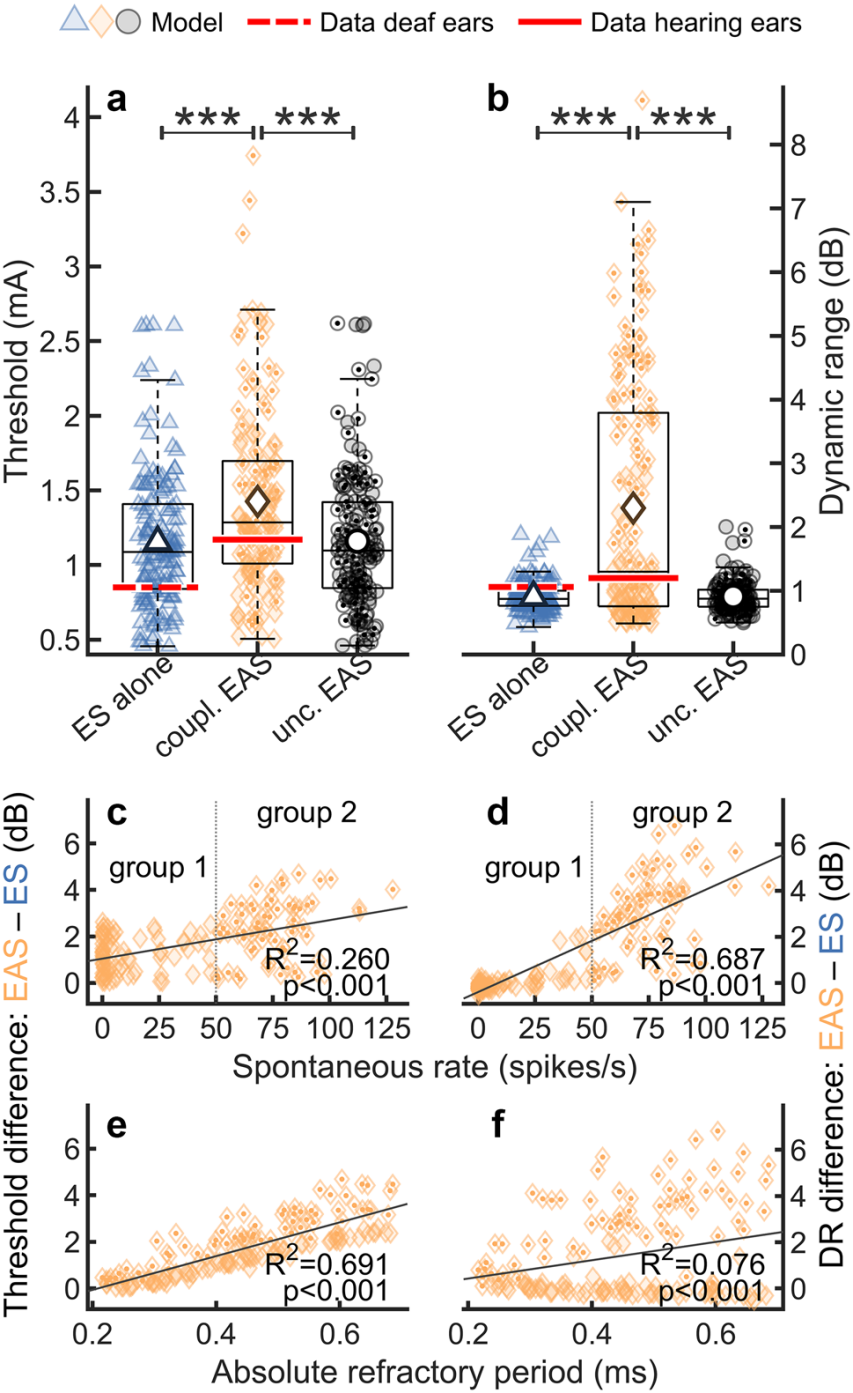
Thresholds **a** and DRs **b** for ES with 40 µs/phase biphasic pulses. Acoustically sensitive ANFs (uncoupled and coupled EAS model) are compared to acoustically insensitive ANFs without spontaneous activity (ES alone model). Population averages are indicated with large markers inside the boxplots. Experimental data of Miller et al. [58] for ANFs from both hearing and deaf ears is indicated with red horizontal lines. The significance of differences between the model predictions was tested using the Mann–Whitney U-test. **c**, **e** Threshold differences between the coupled EAS model and the ES alone model (EAS-ES) as a function of SR and ARP. **d**, **f** DR differences between the coupled EAS model and the ES alone model (EAS-ES) as a function of SR and ARP. Lines indicate linear regression of the data. Markers with a dot indicate ANFs with SR > 50 (group 2)

Figure 3 compares thresholds (a) and DRs (b) across the different models. The results were based on responses to the first pulse of the pulse train for comparison with the data of Miller et al. [58]. ANFs that were simulated with the coupled EAS model (yellow) had increased thresholds when compared to ANFs without spontaneous activity simulated with the ES alone model (blue). The mean predicted threshold for ANFs with spontaneous activity was 1.43 mA (coupled EAS model), whereas the mean predicted threshold for ANFs without spontaneous activity was 1.15 mA (ES alone model), and the mean threshold obtained with the uncoupled EAS model (black) was 1.16 mA. A Mann–Whitney *U*-test ($${n}_{1}={n}_{2}=150)$$ confirmed that the thresholds predicted for ANFs without IHC input (ES alone model) were significantly lower than the predictions for acoustically sensitive ANFs simulated with the coupled EAS model ($$p<0.001$$, $$U=7911$$). This threshold difference was similarly observed in the experimental study of Miller et al. [58] where the mean thresholds of ANFs from hearing and deaf ears were reported as 1.17 mA and 0.85 mA, respectively (red horizontal lines in panel a). The threshold difference between the uncoupled EAS model and the coupled EAS model was also significant ($$p<0.001$$, $$U=7977$$), indicating that the threshold elevation was caused by interaction between spontaneous and electrically evoked activity introduced by the coupling mechanism. No significant difference was found between the uncoupled EAS model and the ES alone model ($$p=0.872$$, $$U=11372$$).

Predicted DRs of ANFs without spontaneous activity represented by the ES alone model (mean DR: 0.90 dB) were significantly lower than predicted DRs for ANFs with spontaneous activity simulated with the coupled EAS model (mean DR:2.30 dB;$p<0.001$, $U=7000$). The same trend was reported in the experimental data (mean DR of acoustically sensitive ANFs: 1.2 dB, mean DR of acoustically insensitive ANFs: 1.06 dB); however, the authors stated that it was not clear whether this difference was statistically significant due to outliers in their data set [58]. Moreover, DRs for the coupled EAS model were significantly higher than DRs obtained with the uncoupled EAS model (mean DR: 0.91 dB; $p<0.001$, $U=6990$). Again, no significant difference was found between the uncoupled EAS model and the ES alone model ($p<0.885$, $U=11359$).

Panels c–f of Fig. 3 investigate the reason for the different thresholds and DRs in ANFs with and without spontaneous activity. We hypothesized that refractoriness after spontaneous spiking partially reduced the excitability to ES in the coupled EAS models, thereby increasing the threshold. This effect would increase with increasing SR and with increasing duration of the ARP and RRP. Moreover, this interaction could affect ES at high stimulation levels stronger than ES at low levels because of higher electrically driven spike rates. This asymmetry could lead to a flatter rate-level curve and thus explain the observed increase of DRs. These mechanisms would not apply to the uncoupled EAS model because of the missing EAS interaction. In c and d, the data is shown as the difference between predictions of the coupled EAS model and the predictions of the ES alone model and plotted as a function of the SR. Panels e and f present the same data as a function of the ARP. Since the ARP and the RRP of each ANF were linearly correlated in the model, the dependence on ARP and RRP was qualitatively the same. There were at least two clusters of ANFs with qualitatively different behaviors, (i) ANFs with low to moderate SR and (ii) ANFs with high SR. Therefore, the ANFs were divided into two groups: group 1 with $\mathrm{SR}\le 50$ and group 2 with $\mathrm{SR}>50$ (c, d). The ANFs of group 2 are indicated with dotted symbols. The thresholds and DRs of the coupled EAS model were significantly increased with respect to the ES alone model for both SR groups; however, the effect was much stronger for the ANFs of group 2. This shows that the threshold and DR differences between the ES alone model and the coupled EAS model were dominated by the HSR fibers, which were most affected by EAS interaction. For ANFs with high SR (group 2), the threshold and DR differences increased also with ARP as expected, indicating that the abovementioned hypothesis may well explain the observed differences between the models for ANFs with $\mathrm{SR}>50$. Surprisingly, the ANFs of group 1 showed a negative trend of DR difference as a function of ARP that cannot be explained by this theory.

**Fig. 4:**
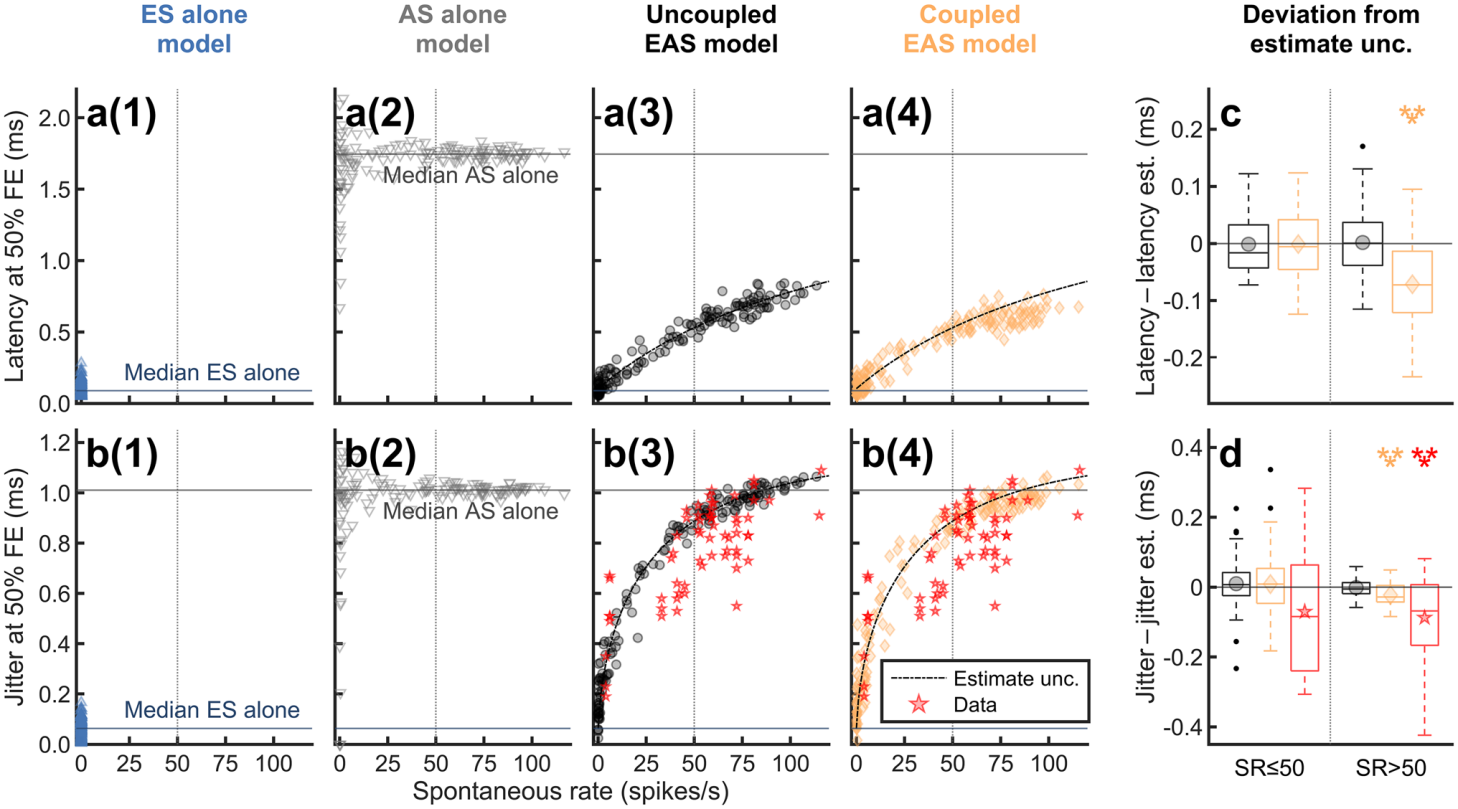
**a**, **b** Latency **a** and jitter **b** for ES with 250 pps pulse trains as a function of SR for the different model variants. All statistics for the ES alone model and the EAS models were obtained at 50 % FE using a time window of 3.5 ms following each pulse. Median latency and jitter of spontaneous activity predicted by the AS alone model (without stimulation) are shown with horizontal lines. Black dashed lines are identical across panels in each row and indicate the analytical estimate for latency (**a3**–**a4**; see Eq. 11) and jitter (**b3**–**b4**; see Eq. 12), assuming no EAS interaction. The predictions for jitter were compared to the experimental data of Miller et al. [58] (red stars). **c**, **d** Deviation of predicted latency **c** and jitter **d** from the corresponding analytical estimate for the data presented in **a3**–**a4** and **b3**–**b4**. The ANFs were divided into two groups based on their SR (SR ≤ 50 and SR > 50). Mean values for each EAS model are indicated by symbols inside the boxplots. Significance was tested using Wilcoxon’s signed rank test

Figure 4 shows the dependency of latency (a) and jitter (b) at the threshold on the SR as predicted by the uncoupled (a3, b3) and the coupled (a4, b4) EAS model. The results of ANFs without spontaneous activity (ES alone model; a1, b1) and of acoustically sensitive ANFs without electric input (AS alone model; a2, b2) are given for comparison. All measures presented in Fig. 4 are based on the spikes occurring in a $T=3.5\; \mathrm{ms}$ time frame directly following each stimulus pulse for comparison with the jitter data of Miller et al. [58] (red stars). In general, the predictions of the uncoupled and the coupled EAS model showed that spontaneous activity increased both latency and jitter. For ANFs without spontaneous activity (ES alone model), the predicted latency ${L}_{deaf}=0.11\; \mathrm{ms}$ and jitter ${J}_{deaf}=0.06\; \mathrm{ms}$ were low. For the other model variants, i.e. in the presence of spontaneous spiking, latency and jitter depended on the length of the observed time frame $T$ (not shown). The expected mean spike latency for spontaneous activity assuming a uniform instantaneous spiking probability is ${L}_{spont}=T/2=1.75\; \mathrm{ms}$ and the expected jitter is ${J}_{spont}=T/\sqrt{12}=1.01\; \mathrm{ms}$ in line with the results of the AS alone model.

Assuming no interaction between spontaneous activity and electrically driven activity in the same ANF (i.e., the uncoupled EAS model), it is possible to analytically estimate latency ($L$) and jitter ($J$) also for ANFs with spontaneous activity as a function of SR (see Appendix 3):

11 $$L=\frac{{N}_{S}}{{N}_{S}+{N}_{E}}\cdot \frac{T}{2}+\frac{{N}_{E}}{{N}_{S}+{N}_{E}}\cdot {L}_{E}\;,$$

12 $$\begin{aligned}J&=\left(\frac{{N}_{S}}{{N}_{S}+{N}_{E}}\cdot \frac{{T}^{2}}{12}+\frac{{N}_{E}}{{N}_{S}+{N}_{E}}\cdot {\left({J}_{E}\right)}^{2}\right.\\&\quad\;\left.+\frac{{N}_{S} {N}_{E}}{{\left({N}_{S}+{N}_{E}\right)}^{2}}\cdot {\left(\frac{T}{2}-{L}_{E}\right)}^{2}\right)^{1/2}\;,\end{aligned}$$

where ${N}_{S}=\mathrm{SR}\cdot T$ is the expected number of spontaneous spikes in the observed time frame $T,$ and ${N}_{E}$ is the expected number of electrically evoked spikes in the same time interval. ${L}_{E}$ and ${J}_{E}$ denote the mean latency and jitter of the electrically driven activity in the absence of spontaneous spiking, respectively. Black dashed lines in panels a3–a4 and b3–b4 show the analytical estimates given by Eqs. (11) and (12) using $T=3.5\; \mathrm{ms}$ and ${N}_{E}=0.5$ for stimulation at the electric threshold.

Panels c and d of Fig. 4 depict the deviations of latency and jitter predicted by the uncoupled and the coupled EAS model from the analytical estimates (11) and (12). Since the analytical estimates were based on the assumption that spontaneous spikes and spikes evoked by ES would not interact, deviations from the estimates indicate an effect of EAS interaction in the model predictions. The ANFs were again divided into two groups based on their SR (group 1: $\mathrm{SR}\le 50$; group 2: $\mathrm{SR}>50$). A Wilcoxon signed rank test was applied to test if the median deviations from the analytical estimate were different from zero. For group 1 ($\mathrm{SR}\le 50)$, none of the EAS models deviated significantly from the analytical estimate. For group 2 ($\mathrm{SR}>50)$, the latency deviations ($p<0.001$, $N=69$) as well as the jitter deviations ($p<0.001$, $N=69$) predicted by the coupled EAS model were significant. This was consistent with the experimental data of Miller et al. [58], which for high $\mathrm{SR}>50$ differed significantly from the analytical jitter estimate, whereas the deviation was not significant for small $\mathrm{SR}\le 50$ (d). In all cases, the predictions of the uncoupled EAS model were identical to the analytical estimates. This result was consistent with the assumption underlying Eqs. (11) and (12) that spontaneous activity and electrically evoked activity would not interact. In contrast, for the coupled EAS model, the statistics demonstrated an impact of the EAS interaction included in this model on latency and jitter for large SRs ($\mathrm{SR}>50$).

### Experiment 3: The Novel EAS Model with Electric-Acoustic Stimulation

The third experiment investigated activity in acoustically sensitive ANFs evoked by simultaneous EAS. The simulations were performed with the uncoupled and the coupled EAS model. The stimulus paradigm was based on the study of Miller et al. [21]. ES was a constant-amplitude pulse train consisting of 40 µs/phase biphasic (cathodic leading) pulses presented at 250 pps. For each ANF, four current levels were randomly selected across the fiber’s DR.

**Fig. 5:**
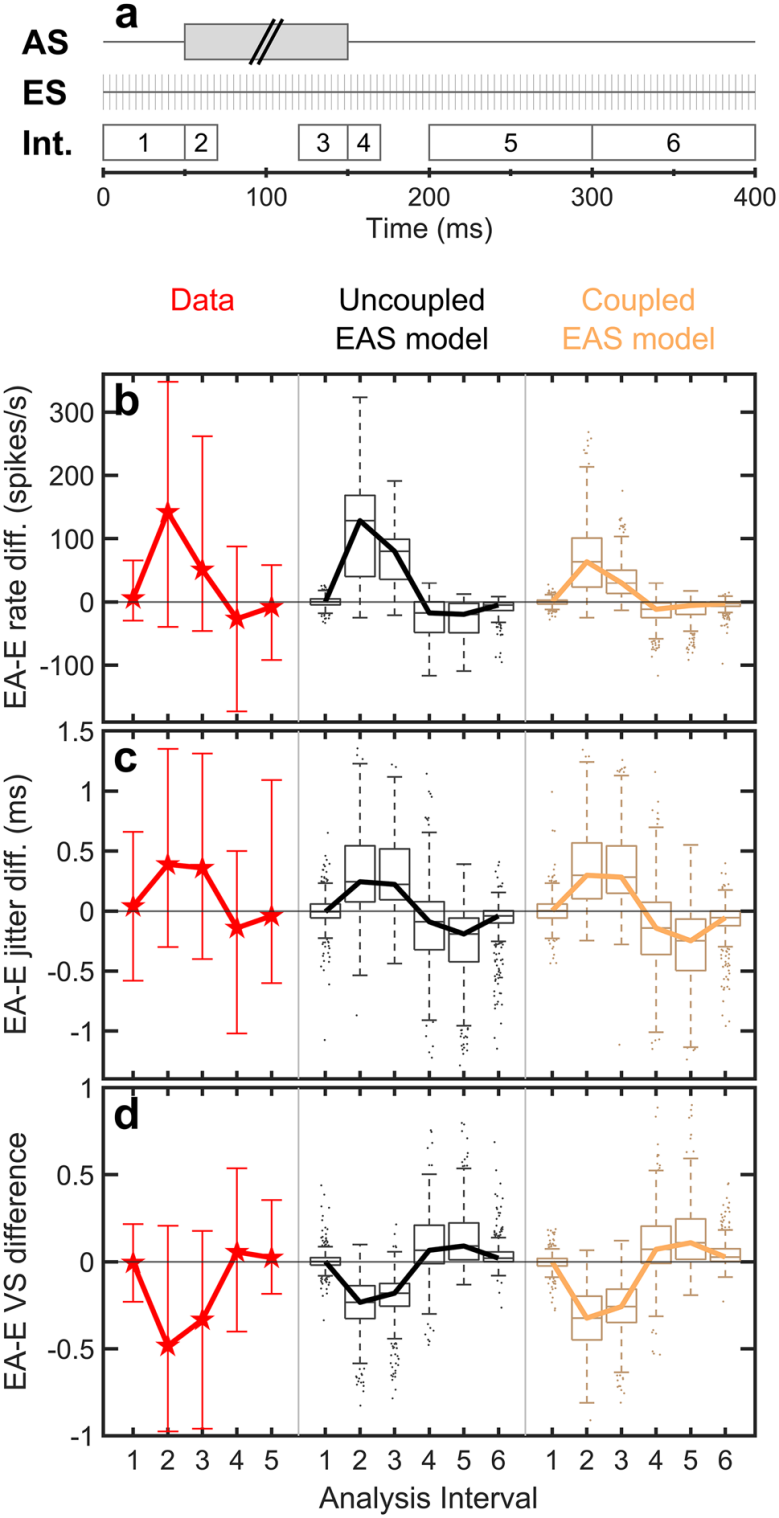
**a** Stimulus paradigm and definition of analysis intervals for experiment 3. The ES pulse train started 50 ms before the AS noise and ended 250 ms after the AS offset. I1 and I2 were defined relative to the AS onset, whereas I3–I6 were defined relative to the AS offset. The experiment was conducted with a mixture of 100 ms, 200 ms, or 300 ms acoustic noise stimuli. **b**–**d** Predictions for the six analysis intervals, pooled across AS duration and ES level. The plots show the “EA-E” differences between the electric + acoustic and the electric-only conditions for spike rate **b**, jitter **c**, and vector strength (VS) **d**. Predictions obtained with the uncoupled EAS model (black) and the coupled EAS model (yellow) are compared to the median experimental data of Miller et al. [21] (red stars). Error bars for the experimental data represent the range of the data set

AS was a broadband noise with a duration of either 100 ms, 200 ms, or 300 ms (1/3 each) presented at levels between 70 and 100 dB SPL. The electric pulse train started 50 ms before the AS onset and ended 250 ms after the AS offset. Six analysis intervals (I1–I6) were defined relative to the AS on- and offset similar to the study of Miller et al. [21] (Fig. 5a). I1 spanned the 50 ms time period before the AS onset. I2 covered the 20 ms following the AS onset. I3 and I4 covered 30 ms before and 20 ms after the AS offset, respectively; and I5 was defined as a 100 ms period starting 50 ms after the AS offset. The rationale was that I1 and I5 should not be influenced by AS, whereas I2–I4 should provide results related to AS on- and offset responses. An additional analysis interval I6 that has not been considered in the experimental study of Miller et al. [21] was defined as the 100 ms period directly following I5.

Panels b-d of Fig. 5 summarize the outcomes of experiment 3 regarding spike rates (b), jitter (c), and VS (d). The predicted data was pooled across the three different AS durations and the four different ES current levels as in the study of Miller et al. [21]. The boxplots show the “EA-E” difference between the data obtained with simultaneous electric + acoustic presentation and the data for electric-only stimulation to assess the effect of the AS noise as a function of the analysis intervals. The medians computed across the ANF population are highlighted with black (uncoupled EAS model) and yellow (coupled EAS model) lines to be compared against the experimental data of Miller et al. [21] (red stars). Error bars for the experimental data depict the range of the data.

In general, the effects associated with the AS noise can be divided into peri-stimulus and post-stimulus effects. During the AS noise (I2 and I3), the spike rate was substantially increased when compared to the ES-only condition. In contrast to the synchronous spiking in response to the ES pulse train, the additional activity evoked by the AS noise consisted of randomly timed spikes thereby increasing jitter and decreasing VS. Following the AS offset (I4), the transient off-suppression of spontaneous spiking in the AS model caused an opposite effect. Spike rates in the electric + acoustic condition were now decreased relative to the electric-only condition, while the absence of the randomly timed spontaneous activity enhanced the synchronicity between spikes. The off-suppression declined with increasing time distance from the AS offset and all measures recovered to their pre-masking state in I5 or I6. This general qualitative behavior was reflected in both model variants as well as the experimental data. Quantitative differences between predictions and data were mainly restricted to two time frames. First, the spike rate in response to the AS noise and especially the strong AS onset response in I2 was underestimated by the coupled EAS model but not by the uncoupled EAS model. Secondly, the predicted recovery from the off-suppression visible in the post-stimulus intervals I4–I6 was slower than reported from the experimental data. For interval I5, the models predicted considerable deviations in jitter and VS between the electric + acoustic and the electric-only conditions, whereas the experimental data at that time showed almost complete recovery from the offset effects. Comparable recovery was predicted by the EAS models for the additional interval I6, showing that the recovery of the EAS models was roughly 100 ms slower than the experimental measurements.

The underestimation of the onset response in interval I2 by the coupled EAS model may have several reasons. Parametric simulations were performed to investigate the effects of (i) increasing the AS stimulation level, (ii) decreasing the impairment of IHCs and OHCs, and (iii) shortening the onset interval I2. Appendix 4 shows that these three parameters can affect the predicted responses of the coupled EAS model to better match the experimental data but that none of the effects examined can by themselves explain the complete difference.

**Fig. 6:**
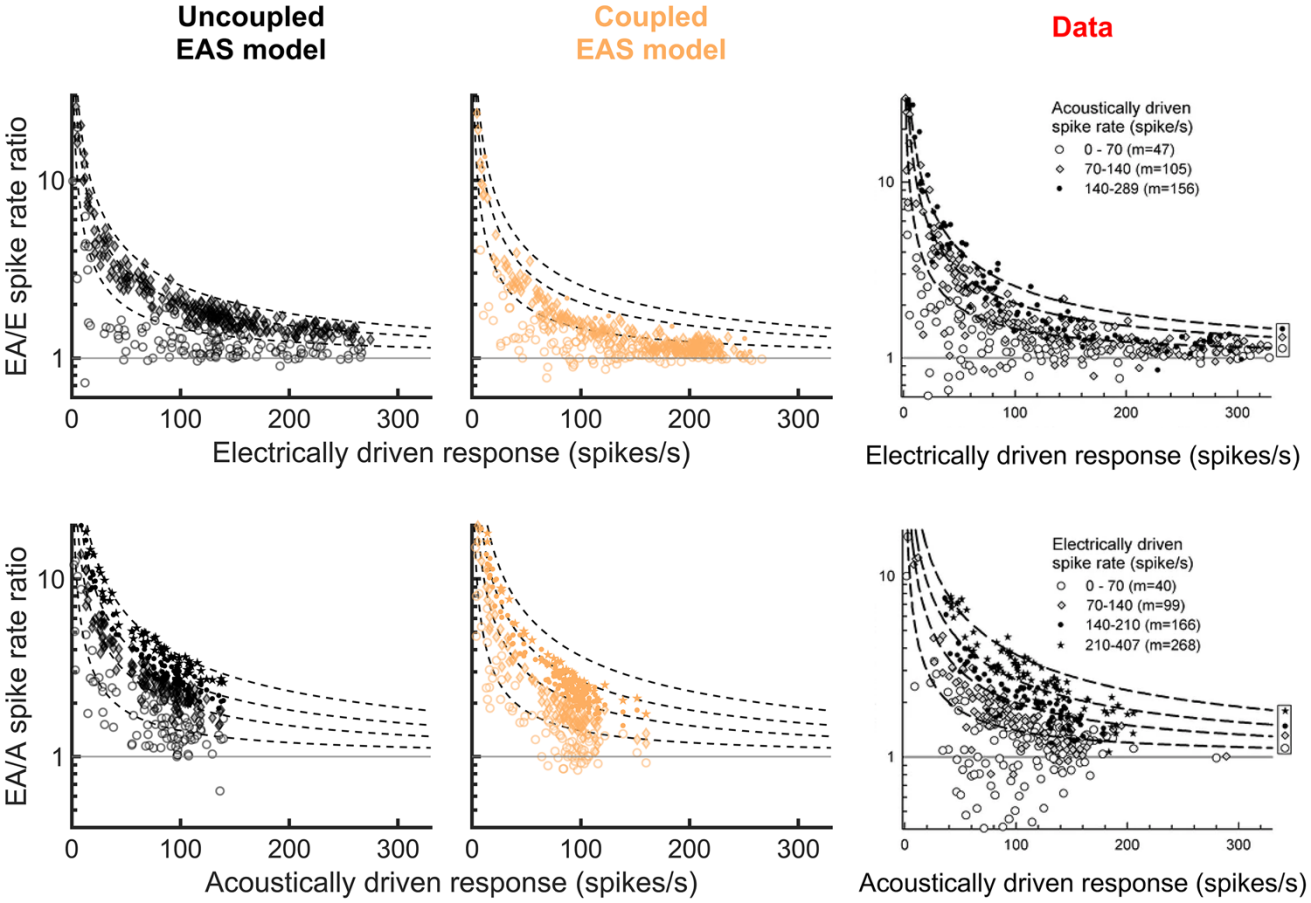
Sustained spike rates measured during analysis interval I3. Top row: ratios of spike rates evoked by the electric + acoustic stimuli (EA) and the electric-only stimulus (E), plotted against the electric-only rate. Bottom row: same as in the top panels, but using the acoustically driven spike rate (A) instead of the electrically driven rate. Predictions of the uncoupled and the coupled EAS models are compared to experimental data of Miller et al. [21]. The ANFs have been grouped according to their acoustically driven (top row) or electrically driven (bottom row) spike rate as indicated in the legends. The dashed lines indicate the theoretical curve for each group median $m$ of the experimental ANF population, assuming linear addition of E + A spike rates. They are identical across the panels in each row, allowing for a better orientation when comparing simulated and experimental results. The data have been reprinted by permission from the Association for Research in Otolaryngology: Springer Nature, JARO - Journal of the Association for Research in Otolaryngology, Miller et al. [21], Copyright (2009)

Figure 6 shows the sustained spike rates derived from interval I3. The predicted rates evoked by the combined electric + acoustic stimuli were normalized and plotted against the rate evoked by the electric-only stimulus (EA/E spike rate ratio, top row) or the acoustic-only stimulus (EA/A spike rate ratio, bottom row). Panels on the right depict the corresponding empirical data of Miller et al. [21]. In that study, the ANFs were grouped according to their acoustically (top) or electrically (bottom) driven spike rate as indicated in the figure legends, where $m$ indicated the median driven spike rate of each group. Different symbols are used to indicate the results for each group. The dashed hyperbolic curves in the “data” panels represent the theoretical curves for the median $m$ of each group of the experimental population, assuming linear addition of E + A spike rates. The same curves were plotted in the other panels to allow for a better orientation when comparing predicted and experimental results. Miller et al. [21] observed suppression between both stimulation modalities especially at high driven response rates, as indicated by most of the points being below their corresponding curve. The coupled EAS model also predicted suppression at large electrically (top) or acoustically (bottom) driven response rates, as indicated by the deviation from the uncoupled EAS model. Thus, the coupled EAS model reproduced the suppression effect observed in the empirical data, in contrast to the uncoupled EAS model. It should be noted that the acoustically driven spike rates in the simulation (bottom) were lower than 150 spikes/s, whereas higher rates of more than 200 spikes/s were achieved in the experimental study. This may again indicate that the effective acoustic stimulation levels were probably lower in the simulation than in the experiment.

## Discussion

### Summary of Main Results

A novel computational model of ANF spiking evoked by ES, AS, or combined EAS was presented. The model is based on the combination of an existing model of ANF activity for AS alone [35] and an extended version of an ANF model for ES alone [42]. An uncoupled, as well as a coupled EAS model, was implemented to investigate EAS interaction mediated by the refractoriness of the ANF.

#### Experiment 1: Extension of the ES Alone Model

Experimental studies on single-ANF responses showed strong interunit variability in the responses to AS or ES (e.g., [22, 53, 60, 61]). The AS model by Bruce et al. [35] reproduces this variability by applying randomized parameters for the refractory behavior, CF, and SR across the ANFs in a fiber population. A similar strategy was applied here to extend the ES model of Joshi et al. [42]. Based on the finding that the electric threshold is determined by the membrane capacitance ($C$), a random distribution of membrane capacitances was constructed to reproduce the thresholds reported for monophasic stimulation (Appendix 1). Secondly, to reproduce the variability in refractory behavior, the parameters affecting the duration of the ARP and RRP in the ES model (${t}_{dead}$ and ${\tau }_{supra}$) were scaled in each individual ANF according to their randomized counterparts from the AS model (${t}_{abs}$ and ${t}_{rel}$). This implied the assumption that ARP as well as RRP in response to AS or ES are correlated in the same ANF. As a direct result of the parameter randomization, the extended ES model realistically reproduced population averages as well as interunit variability in terms of electric thresholds and suprathreshold masking. As an indirect consequence, the extended ES model also predicted reasonable interunit variability for latency, jitter, or subthreshold masking (Fig. 2 and Appendix 2). A similar approach of randomizing the noise amplitudes (${\sigma }_{noise}$) across ANFs to produce more realistic interunit variability in RS was rejected because larger ${\sigma }_{noise}$ led to undesired spontaneous spiking in the ES model. Instead, the noise amplitudes were chosen as fixed values according to Table 1. In consequence, the variance in RS across the simulated ANF population was smaller than what has been reported from animal experiments (Table 3).

#### Experiments 2 and 3: Interaction Between ES, AS, and Spontaneous Activity

Intact IHCs generate spontaneous neurotransmitter releases leading to a wide range of SRs. This spontaneous activity transiently reduces the excitability of the ANF for ES and AS through refractoriness. Experiment 2 investigated the interaction between ES and spontaneous activity. The coupled EAS model predicted increased electric thresholds and DRs in ANFs with spontaneous activity when compared to ANFs from a deaf ear simulated with the ES alone model (Fig. 3) in accordance with experimental findings [58, 62, 63]. These elevations were attributed to the EAS interaction contained in the coupled EAS model, as demonstrated by the uncoupled EAS model, which did not predict increased thresholds or DRs. At the population level, the elevations predicted by the coupled EAS model correlated with SR as well as the duration of the refractory period, which is consistent with the hypothesis that the elevations were caused by (partial) refractoriness of the ANFs after spontaneous spiking. For the subgroup of ANFs with $\mathrm{SR}\le 50$, however, the DR difference between the coupled EAS model and the ES alone model showed a negative trend as a function of ARP, which cannot be explained with this hypothesis. Further experiments with the model or more detailed comparisons with animal data are necessary to understand this phenomenon.

The elevation of thresholds and DRs could potentially affect human EAS users, which may have spontaneous activity in electrically stimulated ANFs around the crossover frequency between ES and AS. In real ANFs, the DR could also be increased due to additional electrophonic responses elicited at low stimulation levels [63, 64]. Electrophonic responses are generated by hair cells and have lower thresholds and larger DR than direct electroneural responses [53, 64, 65]. Although electrophony may have an impact on DR, it is nevertheless unlikely to be the sole cause of the DR increase because (i) the reported thresholds of acoustically sensitive ANFs were increased [58] and not reduced as expected for electrophony; and (ii) the coupled EAS model predicted DR increases purely based on suppressive interaction between electroneural and spontaneous activity, whereas electrophonic excitation was not present in the model.

Experiment 2 also showed that increases in latency and jitter with the SR were rather a statistical result related to the random timing of spontaneous spikes than a consequence of EAS interaction (Fig. 4). An analytical model (Appendix 3) assuming linear addition of spontaneous and electrically evoked spikes largely matched the predictions of both model variants. This shows that in experiment 2, latency and jitter were little affected by EAS interaction. Only at high SRs where the strongest interaction was expected ($\mathrm{SR}\ge 50$), latency and jitter predicted by the coupled EAS model deviated significantly from the analytical estimates and from the uncoupled EAS model. Latency and jitter increased as a function of SR, whereby the two extremes ($\mathrm{SR}=0$ and $\mathrm{SR}\gg 0$) were defined by the ES alone model and the AS alone model, respectively. The analytical model showed that this increase with SR can be understood as a statistical effect of adding randomly timed spontaneous activity to the electrically evoked spike trains. The experimental results of Miller et al. [58] were in good agreement with the jitter predicted by the coupled EAS model, especially when taking into account that the ES alone model slightly overestimated the jitter (Fig. 2c). It would be interesting to perform the same empirical study at different pulse rates or different electric stimulus levels, as it is clear from Eqs. (11) and (12) that latency and jitter depend on the observation time window $T$ and the expected number of electrically evoked spikes ${N}_{E}$ according to the analytical model.

For combined EAS in experiment 3, the EAS models reproduced the qualitative behavior known from experimental data. Experiment 3 revealed strong interaction effects when comparing the coupled EAS model to the uncoupled EAS model. The mutual suppression between spiking evoked by ES and AS were most pronounced around the onset of the acoustic stimulus, i.e. when the highest spike rates were expected. In interval I2 (AS onset), the coupled EAS model predicted “EA-E” spike rate differences reduced by almost 50 % when compared to the uncoupled EAS model. This led to the curious result that the uncoupled EAS model seemed to better match the experimental data than the coupled EAS model. However, this effect was likely caused by differences in the experimental setups. For the experimental measurements, Miller et al. [21] set the acoustic stimulation levels between 70 and 100 dB SPL “to evoke a strong response (…)”. For the simulations, this criterion was defined in terms of a spike rate evoked by AS that exceeded the SR by at least 250 % (maximal 100 dB SPL). It is possible that the effective stimulation levels used by Miller et al. [21] were higher and thus evoked stronger onset responses than the levels used for the simulations. This explanation is supported by Fig. 6, showing that the saturated acoustically driven spike rates (interval I3) in the experimental setup were higher than in the simulations. Increasing the AS noise level by up to 30 dB resulted in a better match between predictions and data, although the predicted onset spike rate was still too low (Appendix 4). It is also possible that differences in hearing status influenced the results. The impairment of IHCs and OHCs in the EAS models (${c}_{IHC}$ and ${c}_{OHC}$) was chosen based on the average hearing loss of 26 dB reported by Miller et al. [21]. However, the authors also reported large within-subject and across-subject variations in acoustic thresholds for a similar preparation, and also that most of the measurements were conducted in a setup with a lower average hearing loss [58]. Thus, the experimental ANF population likely had more variation in the hearing status and probably a generally lower degree of hearing loss than the simulated population. A reduced impairment of the IHCs and OHCs, associated with better acoustic hearing, had a similar effect on the model predictions as an increase in the AS level. Another cause of the weaker onset response in interval I2 predicted by the coupled EAS model could be that the spike rates in the E + A condition considerably decreased across the duration of the interval as a consequence of adaptation. As interval I2 was meant to be short enough to cover only the onset response without significant influence of adaptation, the effect of reducing the duration of interval I2 was investigated. Shorter analysis intervals led to strongly increased spike rates predicted by the coupled EAS model. In conclusion, all of the investigated parameters (AS level, hair cell impairment, interval I2 duration) could influence the model toward a better match between predicted and experimental data. However, each of these effects on its own was too small to overcome the whole mismatch. Therefore, the real reason possibly was a mixture of the investigated effects (Appendix 4). In contrast, the deviations in the post-masking interval I5 were likely caused by a slower recovery of spontaneous activity in the AS model than in the experiment.

### Alternative Coupling Variant

An alternative interaction mechanism between the ES model and the AS model was tested in addition to the coupled EAS model described above. Here, the original setup of the AS model including the spike generator block was preserved. In both the original ES and AS models, the refractoriness of the ANF is implemented as adaptation processes that were triggered upon a spike occurrence. For the alternative EAS coupling, the AS model and the ES model exchanged their spike occurrences at runtime. To mimic the situation that ES and AS simultaneously act on the same ANF, each spike triggered the refractory processes in both models synchronously, such that both systems were set into ARP and RRP irrespective of which model generated the action potential. By this mechanism, the spikes from the AS model suppressed simultaneous electrical activation of the ANF by setting the ES model into a refractory state and vice versa.

Surprisingly, the alternative and the standard coupled EAS models produced almost identical results throughout all experiments shown in this publication. Despite the different coupling approaches, it appeared that both models effectively worked very similarly, a possible explanation being that both approaches were limited to suprathreshold suppressive EAS interaction mediated through the refractoriness of the ANF. A block diagram of the alternative EAS model as well as all figures for experiments 2 and 3 including the predictions from this alternative coupled EAS model can be found in Online Resource 1.

The supplied model code (https://doi.org/10.5281/zenodo.5467990) allows switching between the different coupling variants (uncoupled, standard coupling, alternative coupling) of the EAS model.

### Limitations of the Model

Instead of implementing the threshold variability of the ES population model in terms of different membrane capacitances, in reality, the ES thresholds are determined by anatomical and biophysical properties of the ANF (e.g., axonal diameter, internode lengths, characteristics of ion channels) as well as the electrode-nerve interface (e.g., distance and relative orientation of ANFs and the electric field generated inside the cochlea). A more physiologically accurate model could simulate the 3D voltage distribution in the cochlea (e.g., [66–70]) and simulate the excitability of ANFs in terms of the activating function [71].

The coupled EAS model was limited in the step-like waveform and the suprathreshold amplitude of the postsynaptic current. The suprathreshold amplitude was necessary to obtain similar responses to AS as in the original AS model, where each neurotransmitter release event triggered a spike in the ANF unless the fiber was in a refractory state [35]. As a consequence, by design, the interaction between spikes originating from the acoustic pathway (acoustically evoked or spontaneous activity) and from ES was largely restricted to the suprathreshold regime.

The combination of two existing models for ES and AS provided a simple and robust way of simulating responses to EAS. However, the combined EAS model also inherits some limitations from the underlying base models, for instance, the short latencies predicted by the ES model (Figs. 2b) and the slow recovery of spontaneous spiking from the off-suppression produced by the AS model (Fig. 5). Moreover, the ES model does not simulate electrophonic stimulation. Therefore, the EAS model does not capture possible EAS interaction at the level of hair cells [22, 28], even though electrophony may affect up to $\sim 25 \%$ of the ANFs in animal experiments for pulsatile stimulation [21, 53]. In human EAS users, however, electrophonic stimulation seems unlikely due to their high-frequency hearing loss [34] and has been shown not to contribute to psychoacoustic EAS masking [12]. Therefore, the presented EAS model is likely to simulate the stimulation and interaction modes that are relevant for human EAS subjects.

Very few empirical studies have been conducted on single-ANF activity in response to EAS; therefore, the evaluation of the EAS model was limited. The study of Tillein et al. [22] used sinusoidal ES and could not be used to evaluate the EAS model, since the original ES model was fitted and validated to pulsatile ES only.

### Relation to Existing Models

To the best knowledge of the authors, no other computational single-ANF model of combined EAS has been officially published that takes into account the EAS interaction in the ANF. However, the presented EAS model has similarities to the model used in the PhD thesis of Nourski [72]. In that work, an AS model based on Schroeder and Hall [74] was coupled to a stochastic model of ES based on Bruce et al. [38, 39]. A vesicle release from the AS model generated an excitatory postsynaptic current for the ES model, similar to the coupled EAS model in the manuscript at hand. Instead of the models by Schroeder and Hall [74] and Bruce et al. [38, 39], the present study used the more recently developed model of Bruce et al. [35] and an extension of Joshi et al. [42] which more accurately describe the ANF responses to AS and ES. For instance, the ES model of Joshi et al. [42] consists of two separate sub-neurons to allow for excitation by cathodic as well as anodic currents, whereas the ES model of Nourski [72] could only be activated by cathodic stimulation. Moreover, an important advantage of the novel EAS model is that it can simulate populations of ANFs that differ in CF, SR, refractoriness, or excitability.

The structure and the coupling mechanisms of the EAS models are flexible and could also be used with other ES or AS models. Thus, if more advanced models are developed in the future, the current ES or AS model could be replaced to improve the EAS model.

In the future, it would be beneficial to couple the EAS model to a simulation framework for electrically or acoustically evoked compound action potentials [44–46], electrocochleography [73], or central activity [37, 48–51] to further validate the model and facilitate the translation from the animal model to real patient data [17, 19].

## Conclusion

A phenomenological computational model of the ANF responses to combined EAS was developed. The EAS model consists of two existing models for ES alone or AS alone and provides an uncoupled as well as a coupled model variant to assess EAS interaction in the ANF. In the coupled EAS model, the refractoriness of the ANF leads to a suppressive interaction between electrically and acoustically evoked activity and a sublinear addition of E + A spike rates in accordance with published animal data. The model reproduces the lowering of electrical thresholds and dynamic ranges in deafened ANFs without spontaneous activity and the reduction of phase locking by a second stimulus of the other modality. The presented EAS model forms a basis for future investigations of EAS interactions at the level of the auditory nerve.

### Electronic supplementary material

Below is the link to the electronic supplementary material.

Supplementary file1 (PDF 9278 KB)

## Figures and Tables

**Fig. 7:**
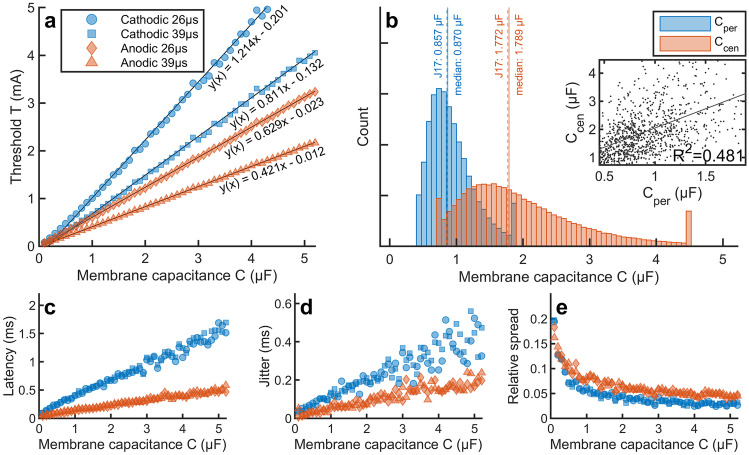
Illustration of the membrane capacitance fitting. **a** Dependency of monophasic thresholds for 26 µs and 39 µs current pulses on the membrane capacitance $C$. Parameters obtained for linear fits are given below each line. **b** Log-normal distributions of the membrane capacitances for the peripheral (blue) and the central neuron (red) constructed based on the linear fits in (a) and the threshold distributions of Miller et al. [47, 55]. Vertical lines show the median parameters across the ANF population (solid, “median”) and the original values used by Joshi et al. [42] (dashed, “J17”). Truncation according to Table 5 is visible as steps at the lower and upper ends of the distributions. The inset shows the correlation R^2^≈0.5 between the membrane capacitances of the peripheral and the central neuron. **c**, **d**, **e** Dependency of latency, jitter, and relative spread on the membrane capacitance. Colors and symbols are identical to (a)

**Fig. 8:**
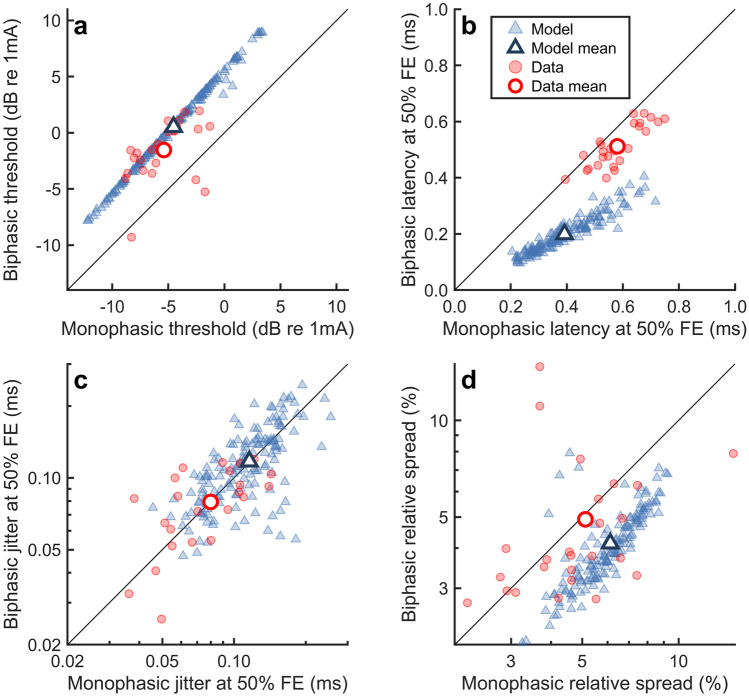
Response characteristics of the ES population model for biphasic cathodic-leading versus monophasic cathodic single pulses with a phase duration of 39 µs. Model outcomes (blue triangles) are compared to experimental data from Miller et al. [57] (red circles). The population averages are shown with large markers. **a** Thresholds. **b** Mean latencies at threshold. **c** Jitter at threshold. **d** Relative spread

**Fig. 9:**
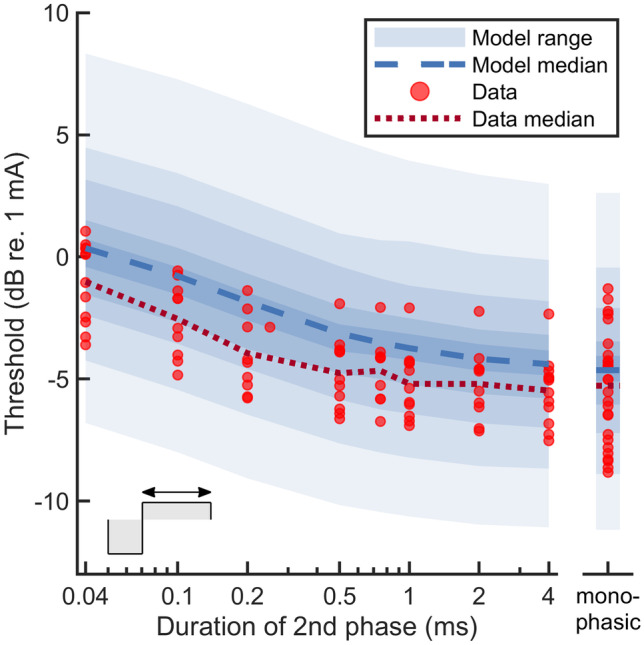
Effect of second-phase duration on single fiber thresholds. The cathodic leading phase had a duration of 40 µs and the amplitude of the anodic phase was adjusted to deliver a net charge of zero. The predicted range (shaded area) and median (blue dashed line) of the ANF population is compared to experimental data of Miller et al. [57] (red circles and dotted line). The different shades of blue indicate quantiles of the simulated data in 11.1 % steps

**Fig. 10:**
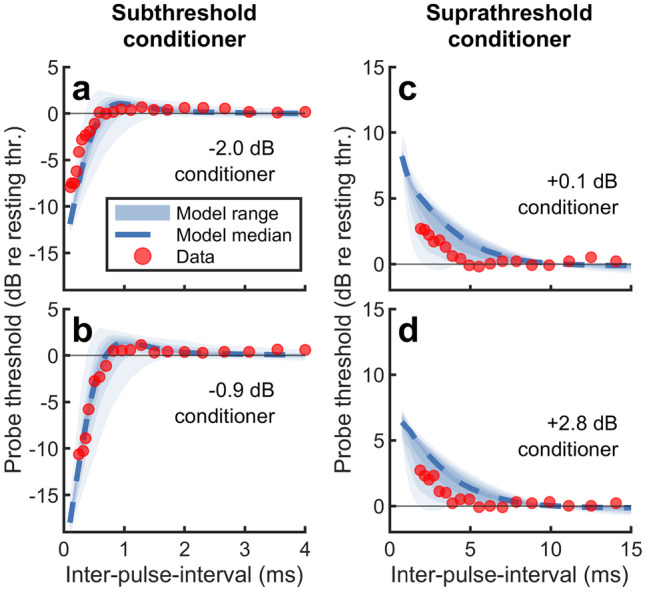
Influence of a subthreshold (**a**, **b**) or suprathreshold (**c**, **d**) conditioner on probe thresholds for different IPIs, evaluated in a forward-masking paired-pulse paradigm. Both conditioner and probe were 100 µs monophasic cathodic pulses and conditioners were presented at -2.0 dB **a**, -0.9 dB **b**, + 0.1 dB **c**, or + 2.8 dB **d** relative to the resting threshold of each ANF. The range (blue shaded area) and the median (blue dashed line) of the masked probe thresholds obtained across the ANF population are compared to experimental data from a single ANF [56] (red circles). The different shades of blue indicate quantiles of the simulated data in 11.1 % steps

**Fig. 11:**
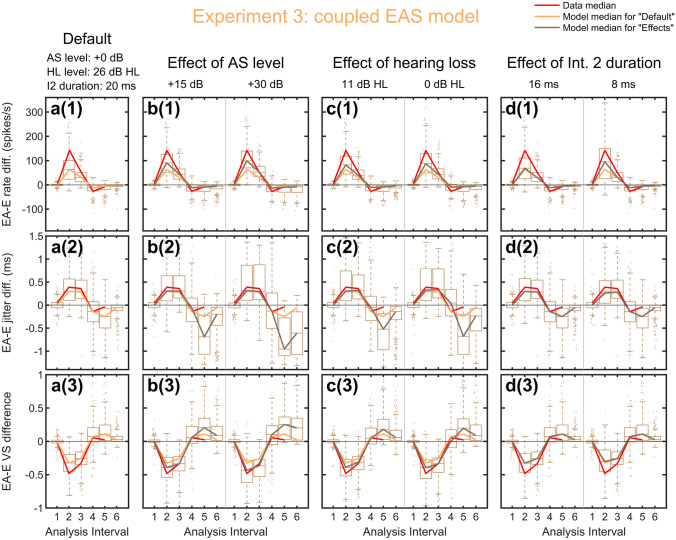
**a** Summarized predictions of the coupled EAS model for experiment 3 as shown in Fig. 5 in the main text. The red line represents the median of the experimental data. **b**–**d** Effect of AS level **b**, HL level **c**, and duration of analysis interval I2 **d** on the predictions of the coupled EAS model. The medians of the experimental data and the “default” simulation are depicted with red and yellow lines, respectively

## Data Availability

The data shown in the figures are available from the corresponding author upon appropriate request.

## Appendix 1

### Parameter randomization and fitting of the ES population model

The ES model by Joshi et al. [42] was originally built upon a fixed parameter set to reproduce the responses of an “average” ANF. Because of this design, the model could not account for across-fiber variability observed in ANF populations. The acoustic part of the EAS model mimics variability across ANFs using randomized parameters for the CF, the SR, and the refractory time constants (see “Methods and Materials - AS Model”). To include across-unit variability also in the electric part of the EAS model, the parameter set proposed by Joshi et al. [42] was reworked and partly randomized. The new, randomized parameter set represented an ANF population with single-fiber thresholds that mimic the physiological mean and variance in ANF thresholds for monophasic stimuli [55].

Figure 7a shows the dependency of thresholds for cathodic stimuli on the membrane capacitance of the peripheral neuron and the dependency of anodic thresholds on the membrane capacitance of the central neuron. The predicted thresholds depended approximately linearly on the membrane capacitance $C$ (because the right-hand side of Eq. (2) is dominated by ${I}_{stim}$), whereas the dependency on other model parameters was more complicated (not shown). Therefore, it was most convenient to randomize only the membrane capacitances, while other parameters (e.g., membrane conductance ${g}_{L}$ or slope factor $\Delta_T$) were kept fixed. The membrane capacitance $C$ also affected latency, jitter, and RS (Fig. 7c-e), hence it was not possible to fit all these interrelated statistics only by changing the membrane capacitance. Similar to Joshi et al. [42], out of these four statistics, the thresholds were selected to determine the membrane capacitance. The threshold curves were fitted with linear models

13 $$T\left(C\right)=m\cdot C+b\;,$$

where $T$ was the threshold in mA, $m$ and $b$ were fitting parameters, and $C$ was the membrane capacitance ${C}_{per}$ of the peripheral neuron (for cathodic thresholds) or ${C}_{cen}$ of the central neuron (for anodic thresholds). The parameter distribution of the membrane capacitances $C$ was chosen such that the corresponding threshold distribution matches the experimental data of Miller et al. [55].

Miller et al. [55] reported thresholds (defined as the stimulus level evoking a FE of 50 %) in dB for 26 µs and 39 µs monophasic pulses of both polarities. Assuming a Gaussian distribution of thresholds on the logarithmic dB scale, the thresholds needed to be log-normally distributed on a linear ampere scale. This was achieved by setting the distribution of membrane capacitances to

14 $$C={10}^{\mu +\sigma x}-b/m\;,$$

where $x$ was a normally distributed random variable, $b$ and $m$ were the fitting parameters for the threshold curves ${T}(C)$ stated in Fig. 7a, and $\mu$ and $\sigma$ were parameters that needed to be optimized to obtain the desired mean and variance of the threshold distributions. Calculating the resulting distribution of thresholds in dB based on Eqs. (13) and (14), we get

15 $$\begin{aligned}T\,\left[dB\right]&=20\log_{10}\left(mC+b\right)=20\log_{10}\left(m10^{\mu+\sigma x}\right)\\&=\underbrace{20\left(\log_{10}\left(m\right)+\mu\right)}_{\mathrm M}+\underbrace{20\;\sigma}_\triangle\;x=\mathrm M+\Delta x\end{aligned}$$

where the mean $\mathrm{M}$ and the standard deviation $\Delta$ of $T$ in dB were known from the experiments of Miller et al. [55]. According to Eq. (15), the parameters $\mu$ and $\sigma$ of the membrane capacitance distribution in Eq. (14) are

16 $$\mu =\frac{\mathrm{M}}{20}-{\mathrm{log}}_{10}\left(m\right)\;\mathrm{and}\;\sigma =\frac{\Delta }{20}.$$

The resulting values of $\mu$, $\sigma$, and $b/m$ for cathodic and anodic stimulation with 26 µs and 39 µs pulses are listed in Table 4. The parameters were averaged across the two pulse durations in order to obtain the final values for the distributions of ${C}_{per}$ and ${C}_{cen}$. The random variables $x$ of the peripheral and the central neuron were constructed with a correlation of ${R}^{2}=0.5$ between the two random variables in order to restrict the combination of high capacitances for the peripheral neuron with low capacitances of the central neuron of the same ANF, and vice versa.

**Table 4:** Population parameters derived for the distribution of membrane capacitances

**Stimulus**	$\mathbf{M}$ ^a^	${\varvec{\Delta}}$ ^a^	$-{\varvec{b}}/{\varvec{m}}$ **(nF)** ^b^	${\varvec{\mu}}$ ^c^	${\varvec{\sigma}}$ ^c^
*Cathodic 26 µs*	**–0.88**	**2.99**	**165.7**	**–6.1283**	**0.1495**
*Cathodic 39 µs*	**–5.31**	**4.80**	**162.3**	**–6.1745**	**0.2400**
Average peripheral neuron			**164.0**	**–6.1514**	**0.1947**
*Anodic 26 µs*	**1.00**	**3.64**	**36.8**	**–5.7489**	**0.1820**
*Anodic 39 µs*	**–2.72**	**4.40**	**28.5**	**–5.7604**	**0.2200**
Average central neuron			**32.7**	**–5.7547**	**0.2010**

^a^From Miller et al. [55]

^b^from linear fit in Fig. 7a

^c^derived using Eq. (16)

In order to avoid extraordinarily low or high membrane capacitances, which may lead to unstable simulations, the unbounded distributions in Eq. (14) were truncated at ${C}_{min}={10}^{\mu -2\sigma }-b/m$ and ${C}_{max}={10}^{\mu +2\sigma }-b/m$. The resulting limits for both neurons are listed in Table 5. The final distributions of peripheral and central membrane capacitances are shown in Fig. 7b.

**Table 5:** Limits for the distributions of membrane capacitances

**Parameter**	**Minimum ** $[\mathbf{n}\mathbf{F}]$	**Maximum ** $[\mathbf{n}\mathbf{F}]$
${C}_{per}$	$341.3$	$2293.6$
${C}_{cen}$	$591.5$	$4891.6$

The standard deviations ${\sigma }_{noise}$ of the noise currents ${I}_{noise}$ entering the neuronal membrane in Eq. (2) primarily affected the RS of the fiber. Therefore, ${\sigma }_{noise}$ was determined following the fitting of membrane capacitances to best reproduce the mean RS reported by Miller et al. [55].

## Appendix 2

### Additional Simulation Results for Experiment 1

This appendix contains additional simulations to show the effect of parameter randomization on the predictions of the ES alone model, as well as to validate the new implementation of this extended model.

Figure 8 shows how the ES model generalized from monophasic to biphasic stimulation. The subplots show the same measures as in Fig. 2, but for biphasic cathodic-anodic vs. monophasic cathodic pulses with a phase duration of 39 µs. The model reproduced the experimental outcomes for thresholds (a), jitter (c), and RS (d), whereas the predicted mean spike latency (b) for biphasic stimulation was again lower than the experimental results [57]. The predictions for biphasic and monophasic thresholds were strongly correlated (${R}^{2}=0.998$, $p<0.001$) as both depended linearly on the membrane capacitance $C$ (not shown). The experimental data exhibited more variation between biphasic and monophasic thresholds. However, Miller et al. [57] remarked that the three ANFs with lower monophasic than biphasic thresholds produced atypical responses based on RS, anodic vs. cathodic thresholds, and post-stimulus time histograms, and could therefore be regarded as outliers.

The charge integration properties of the ES model were validated based on the thresholds to asymmetric biphasic pulses with a 40 µs leading cathodic phase. Figure 9 shows the thresholds as a function of the duration of the second anodic phase. For each second-phase duration, the amplitude of the anodic phase was adjusted to ensure charge-balanced stimulation. As the duration of the second phase increased, its influence on the threshold decreased, and the biphasic thresholds converged toward their monophasic equivalents. The strongest decline of thresholds was predicted for second-phase durations below 0.5 ms. The model predictions agreed well with experimental data of Miller et al. [57] up to a small absolute offset of about 1 dB.

Figure 10 presents the outcomes of a forward-masking experiment with 100 µs monophasic cathodic pulses. The experiment was conducted with a paired-pulse paradigm, using one fixed-level pulse as the conditioner and a second variable-level pulse as the probe. The inter-pulse interval (IPI) was defined as the time between the conditioner onset and the probe onset. The masked probe thresholds were measured at various IPIs. Probe thresholds were either simulated for preceding subthreshold conditioners or suprathreshold conditioners. For the subthreshold condition, the conditioner level was set below the threshold, and additionally, all simulation runs where the conditioner evoked a spike were rejected. In contrast, for the suprathreshold condition the conditioner level was set above the fiber’s threshold, and runs were rejected if the conditioner did not evoke a spike.

Following a subthreshold conditioner (Fig. 10a, b), probe thresholds were strongly decreased for short IPIs (< 0.8 ms), a phenomenon known as facilitation or summation. The amount of facilitation was related to the level of the conditioner, with higher conditioner levels causing stronger decreases in probe thresholds. Following the facilitation period, the probe thresholds were typically increased beyond the unmasked single pulse threshold. This phenomenon has been referred to as accommodation. The probe thresholds had their maximum for IPIs of about 1.0 ms and decayed toward the single pulse threshold for IPIs between 1.0 and 4.0 ms. Some ANFs showed a prolonged duration of facilitation up to 1.5 ms followed by very low or no accommodation, as indicated by the blue shaded area. The range of thresholds of the simulated ANF population (blue shaded area) fitted well with the experimental data of a single ANF measured by Dynes [56] (red circles) as well as with the predictions of the original ES model by Joshi et al. [42] (not shown).

In the suprathreshold condition (Fig. 10c, d), the conditioner was always followed by an ARP with a duration between 0.2 and 0.7 ms (Table 2). During the ARP, the ANF could not spike irrespective of the stimulus level. The ARP was followed by the RRP characterized by temporarily increased probe thresholds that decay toward the unmasked single pulse threshold within 5 to 15 ms following the conditioner pulse. The experimental data of Dynes [56] (red circles) were within the range of the predicted probe thresholds indicated by the blue shaded area, although the medians of the simulated ANF population showed slightly stronger threshold increases than the experimental data. For both predicted and experimental data, the elevation of probe thresholds was almost independent of the conditioner level.

## Appendix 3

### Analytical Estimation of Latency and Jitter for ES with Spontaneous Activity

Let ${X}_{S}$ and ${X}_{E}$ be two random variables with probability density functions ${f}_{S}$ and ${f}_{E}$. We denote the expectation values with ${\mu }_{S}$, ${\mu }_{E}$ and the corresponding variances with ${\sigma }_{S}^{2}$, ${\sigma }_{E}^{2}$, respectively. In the following, the random variables will represent spike times of spontaneous (*S*) and electrically driven (*E*) activity in an ANF, thus the expectation values and variances will correspond to latency and (squared) jitter.

We assume a joint random variable $X$, where we either pick from ${X}_{S}$ with a probability ${p}_{S}$ or from ${X}_{E}$ with a probability ${p}_{E}=1-{p}_{S}$, i.e. the probability density function of $X$ is $f={p}_{S}{f}_{S}+{p}_{E}{f}_{E}$. $X$ reflects a spike train, which consists of spontaneous and electrically evoked events. The expectation value of the joint variable $X$ is

17 $$\mu ={p}_{S}{\mu }_{S}+{p}_{E}{\mu }_{E}\;,$$

and the variance of $X$ is

18 $$\begin{aligned} {\sigma }^{2} &= \int {\left(x-\mu \right)}^{2}f\left(x\right) \mathrm{d}x\\ &={p}_{S}\int {\left(x-\mu \right)}^{2}{f}_{S}\left(x\right) \mathrm{d}x +{p}_{E}\int {\left(x-\mu \right)}^{2}{f}_{E}\left(x\right) \mathrm{d}x\\ &={p}_{S}\int {\left(\left(x-{\mu }_{S}\right)+\left({\mu }_{S}-\mu \right)\right)}^{2}{f}_{S}\left(x\right) \mathrm{d}x\\ &\phantom{=}\;+{p}_{E}\int {\left(\left(x-{\mu }_{E}\right)+\left({\mu }_{E}-\mu \right)\right)}^{2}{f}_{E}\left(x\right) \mathrm{d}x\\ &={p}_{S}{\sigma }_{S}^{2}+{p}_{S}{\left({\mu }_{S}-\mu \right)}^{2}+{p}_{E}{\sigma }_{E}^{2}+{p}_{E}{\left({\mu }_{E}-\mu \right)}^{2}\\ &={p}_{S}{\sigma }_{S}^{2}+{p}_{S}{\left({p}_{E}{\mu }_{S}-{{p}_{E}\mu }_{E}\right)}^{2}+{p}_{E}{\sigma }_{E}^{2}+{p}_{E}{\left({p}_{S}{\mu }_{E}-{p}_{S}{\mu }_{S}\right)}^{2}\\ &={p}_{S}{\sigma }_{S}^{2}+{p}_{E}{\sigma }_{E}^{2}+{p}_{S}{p}_{E}{\left({\mu }_{S}-{\mu }_{E}\right)}^{2}. \end{aligned}$$

We now assume an ANF with spontaneous spiking (spike times ${X}_{S}$) and electrically driven activity (spike times ${X}_{E}$), where both activities do not interact with each other (linear addition of spike rates). The weights ${p}_{S}$ and ${p}_{E}$ correspond to the probabilities that a given spike in the ANF resulted from the respective spiking mode (*S* or *E*) and depend on the average number of spikes expected for both modes:

19 $${p}_{S}={N_S}/{N},\;{p}_{E}={N}_{E}/N\;,$$

where $N={N}_{S}+{N}_{E}$ is the expected total number of spikes in the observed time interval $T$ and ${N}_{S}=\mathrm{SR}\cdot T$. The number of electric spikes (${N}_{E}$) depends on the electric stimulus level.

Following Eq. (17), the expected latency of spiking in the ANF is

20 $$L=\frac{{N}_{S}}{N}\cdot \frac{T}{2}+\frac{{N}_{E}}{N}\cdot {L}_{E}\;,$$

where ${L}_{E}$ denotes the mean latency of electrically evoked spikes. Similarly, jitter can be estimated using Eq. (18):

21 $${J}^{2}=\frac{{N}_{S}}{N}\cdot {\left(\frac{T}{\sqrt{12}}\right)}^{2}+\frac{{N}_{E}}{N}\cdot {\left({J}_{E}\right)}^{2}+\frac{{N}_{S} {N}_{E}}{{N}^{2}}\cdot {\left(\frac{T}{2}-{L}_{E}\right)}^{2},$$

with ${J}_{E}$ denoting electrically evoked jitter.

For a pulse train presented at electric threshold as in experiment 2, ${N}_{E}=0.5$ evoked spikes are expected in each inter-pulse interval $T$. The simulations for experiment 2 have been performed with a pulse train at 250 pps, i.e. an inter-pulse interval of 4 ms. However, only the spikes in the first 3.5 ms were considered for the analysis to allow comparison to the experimental data (${N}_{S}=\mathrm{SR}\cdot 3.5\,\mathrm{ms}$). It is important to note that this reduced time window did not affect the observation of electrically evoked spikes because electrically driven activity occurred at latencies below 1 ms. Therefore, ${N}_{E}=0.5$ was used for the plots shown in Fig. 4.

## Appendix 4

### Parametric Studies for Experiment 3

Figure 11 investigates possible reasons for the underestimation of the onset response in interval I2 by the coupled EAS model.

First, this deviation may be related to differences in AS stimulation levels between experimental and predicted data, since Miller et al. [21] only generally reported AS levels in the range of 70–100 dB SPL. For the simulations in experiment 3, the same range of levels was used by ensuring that for each ANF the acoustically driven spike rate was at least 250 % of the SR. However, since the exact procedure used to select the levels in the study of Miller et al. [21] was unknown, it is possible that the average stimulation levels used for the simulations were too low. Panels b1–b3 show the median response predicted by the coupled EAS model when the level of the AS noise was increased by + 15 dB or + 30 dB relative to the default level. Larger AS amplitudes resulted in a better match between the coupled EAS model and the experimental data in interval I2 (rate and VS) but also in a stronger offset suppression in interval I5 (jitter and VS).

Secondly, the impairment of inner and outer hair cells defined by the parameters ${c}_{IHC}$ and ${c}_{OHC}$ of the AS model influences the responsiveness to the acoustic noise. In the default condition, ${c}_{IHC}$ and ${c}_{OHC}$ were determined based on a flat hearing loss profile of 26 dB HL. Panels c1–c3 show that reducing the amount of hearing loss to 16 dB HL or to 0 dB HL produced a similar effect on median responses as an increase in the AS level.

Thirdly, it was observed that the onset spike rate of the EAS models significantly declined across the duration of the 20 ms interval I2. This may be a hint that the predicted adaptation of the onset response could be faster than in the experimental ANFs. For panels d1–d3, the duration of interval I2 was therefore reduced from 20 to 16 ms or 8 ms such that it was less affected by adaptation. The predicted onset response was increased for the shorter onset intervals.

To summarize the parametric simulations, all three effects that were investigated could partially account for the underestimation of the experimental data in the onset interval I2, but none of them explains the complete mismatch. It may be concluded that a combination of different effects could have caused the deviation between experimental and predicted medians in interval I2.

## Code Availability

The code for the presented EAS model is available at Zenodo (https://doi.org/10.5281/zenodo.5467990) and GitHub (https://github.com/APGDHZ/Single-fiber-EAS-model/tree/v1.0.0).
