# Supplementary material for: A Computational Model of a Single Auditory Nerve Fiber for Electric-Acoustic Stimulation
Source: J Assoc Res Otolaryngol. 2022 Nov 4;23(6):835–58. doi: 10.1007/s10162-022-00870-2 (PMC9789289; doi:10.1007/s10162-022-00870-2)
Supplement: Supplementary file 1 — Supplementary file1 (PDF 9278 KB) [file 10162_2022_870_MOESM1_ESM.pdf]

Online Resource 1 for:

A computational model of a single auditory nerve fiber for electric-acoustic stimulation

Published in the Journal of the Association for Research in Otolaryngology (JARO), 2022

Daniel Kipping,[a,b,*] Waldo Nogueira[a,b]

[a] *Department of Otolaryngology, Hannover Medical School, Hannover, Germany*

[b] *Cluster of Excellence Hearing4all, Germany*

[*] *Corresponding author: kipping.daniel@mh-hannover.de*

Online Resource 1

This online resource contains additional information and data for the alternative EAS model described in the discussion. The alternative EAS model uses a different coupling mechanism than the coupled EAS model described in the main text. The results presented in this online resource show that the outcomes of the coupled EAS model and the alternative EAS model are almost equivalent for the experiments 2 and 3 conducted in the present study.

This online resource contains the following figures, in brackets the corresponding figure from the main manuscript:

- Figure ESM1: Block diagram and explanation of the alternative EAS model. (→ Figure 1)
- Figure ESM2: Additional results for experiment 2. (→ Figure 3)
- Figure ESM 3: Additional results for experiment 3. (→ Figure 4)
- Figure ESM 4: Additional results for experiment 3. (→ Figure 5)
- Figure ESM 5: Additional parametric analysis for experiment 3. (→ Figure 11)
- Figure ESM 6: Additional results for experiment 3. (→ Figure 6)

The results supplied in this online resource are presented without further discussion. A general discussion of the different model variants is contained in the discussion section of the main manuscript.

The model code for the alternative EAS model as well as for the uncoupled and coupled EAS models is available at Zenodo (https://doi.org/10.5281/zenodo.5467990) and GitHub (https://github.com/APGDHZ/Single-fiber-EAS-model/tree/v1.0.0).

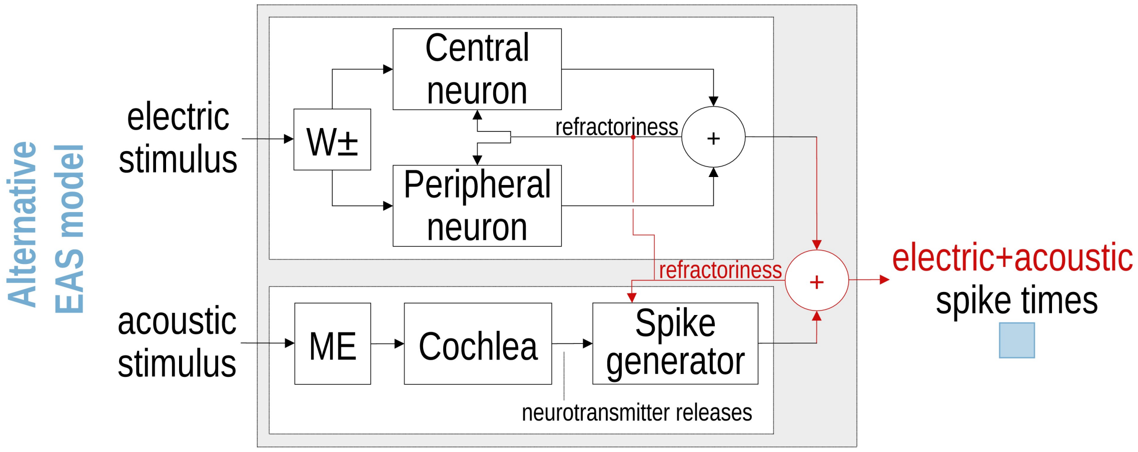

Figure ESM 1. Block diagram of the alternative EAS model. Corresponds to Figure 1 from the main manuscript.

In the ES model as well as in the synapse and spike generator block of the AS model, the refractoriness of the ANF is implemented as adaptation processes that are triggered upon a spike occurrence. In the alternative EAS model, both models exchange information about their spike occurrences at runtime. This information is used to trigger the refractory processes in both models synchronously after each action potential, i.e. the post-spike adaptation processes are applied to both models, irrespective of whether the spike was generated by the ES model or by the AS model.

W± – stimulus weighting block;
Central/Peripheral neuron – adaptive integrate-and-fire point neuron models with sub- and suprathreshold feedback currents;
ME – middle ear filter;
Cochlea – includes basilar membrane, outer hair cells, inner hair cells, and the synapse;
Spike generator – generates spikes by accounting for the refractoriness of the ANF.

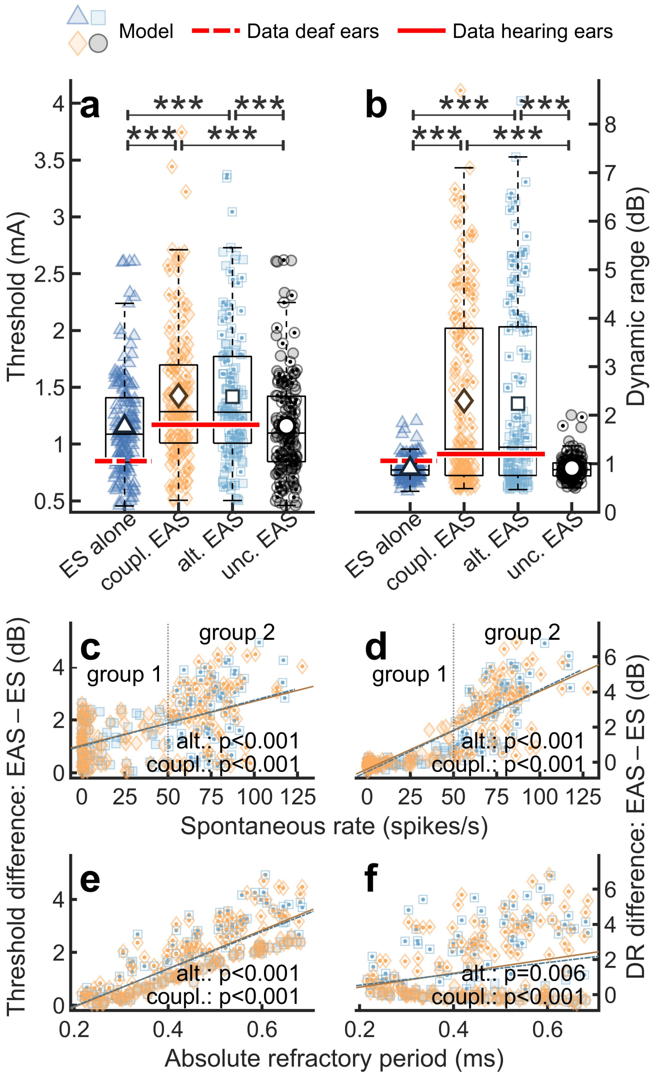

Figure ESM 2. Additional results for experiment 2. Corresponds to Figure 3 from the main manuscript.

Thresholds (**a**) and DRs (**b**) for ES with 40 µs/phase biphasic pulses. Acoustically sensitive ANFs (uncoupled, coupled, and alternative EAS model) are compared to acoustically insensitive ANFs without spontaneous activity (ES alone model). Population averages are indicated with large markers inside the boxplots. Experimental data of Miller et al. (2006) for ANFs from both hearing and deaf ears is indicated with red horizontal lines. Significance of differences between the model predictions was tested using the Mann-Whitney U-test.
c, **e** Threshold differences between the coupled and alternative EAS models and the ES alone model (EAS – ES) as a function of SR and ARP.
d, **f** DR differences between the coupled and alternative EAS models and the ES alone model (EAS – ES) as a function of SR and ARP. Lines indicate linear regression of the data. Markers with a dot indicate ANFs with $\mathbf{SR > 50}$ (group 2).

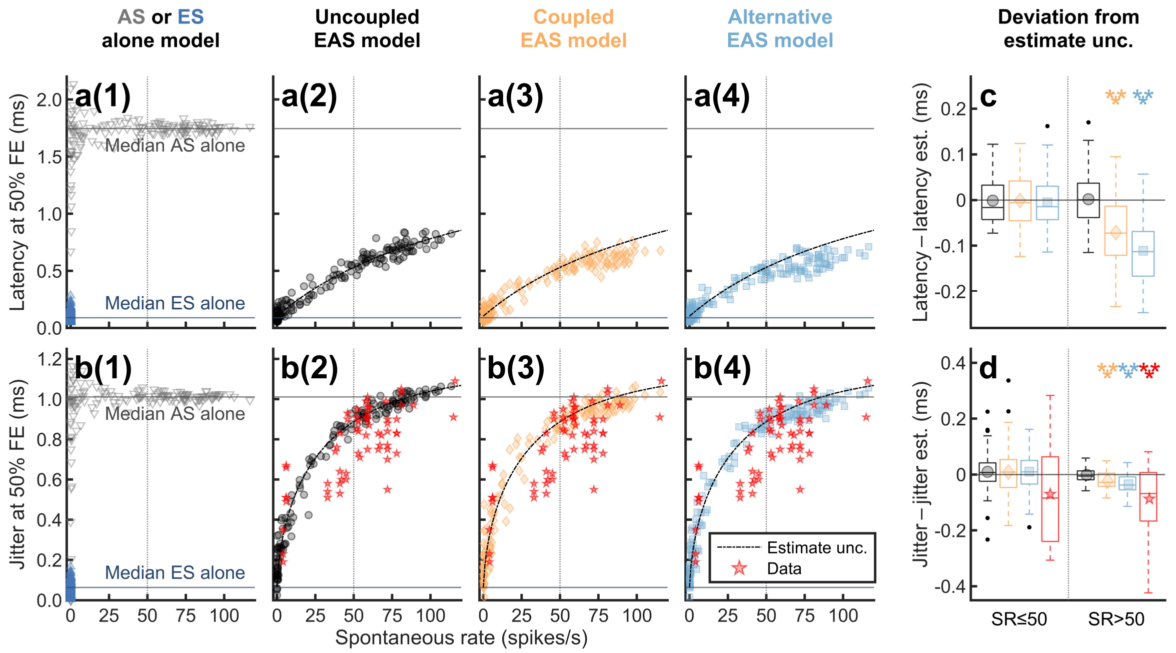

Figure ESM 3. Additional results for experiment 3. Corresponds to Figure 4 from the main manuscript.

a, **b** Latency (**a**) and jitter (**b**) for ES with 250 pps pulse trains as a function of SR for the different model variants. All statistics for the ES alone model and the EAS models were obtained at 50% FE using a time window of 3.5 ms following each pulse. Median latency and jitter of spontaneous activity predicted by the AS alone model (without stimulation) are shown with horizontal lines.
Black dashed lines are identical across panels in each row and indicate the analytical estimate for latency (**a2-a4**; see equation 11 in the main manuscript) and jitter (**b2-b4**; see equation 12 in the main manuscript), assuming no EAS interaction. The predictions for jitter were compared to the experimental data of Miller et al. (2006) (red stars).
c, **d** Deviation of predicted latency (**c**) and jitter (**d**) from the corresponding analytical estimate for the data presented in **a2-a4** and **b2-b4**. The ANFs were divided into two groups based on their SR (SR≤50 and SR>50). Mean values for each EAS model are indicated by symbols inside the boxplots. Significance was tested using Wilcoxon's signed rank test.

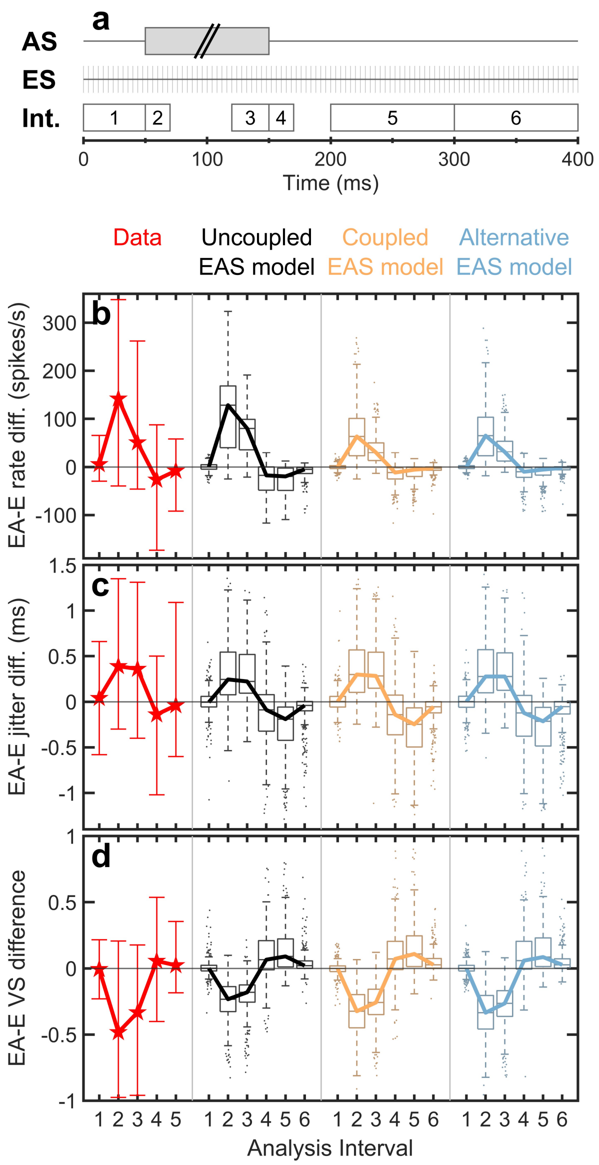

Figure ESM 4. Additional results for experiment 3. Corresponds to Figure 5 from the main manuscript.

a Stimulus paradigm and definition of analysis intervals for experiment 3. The ES pulse train started 50 ms before the AS noise and ended 250 ms after the AS offset. I1 and I2 were defined relative to the AS onset, whereas I3 – I6 were defined relative to the AS offset. The experiment was conducted with a mixture of 100 ms, 200 ms, or 300 ms acoustic noise stimuli.
b-d Median statistics for the six analysis intervals, pooled across AS duration and ES level. The plots show the "EA-E" differences between the electric+acoustic and the electric-only conditions for spike rate (**b**), jitter (**c**), and vector strength (VS) (**d**).
Predictions obtained with the uncoupled EAS model (black), the coupled EAS model (yellow), and the alternative EAS model (light blue) are compared to experimental data of Miller et al. (2009; red stars). Error bars for the experimental data represent the range of the data set.

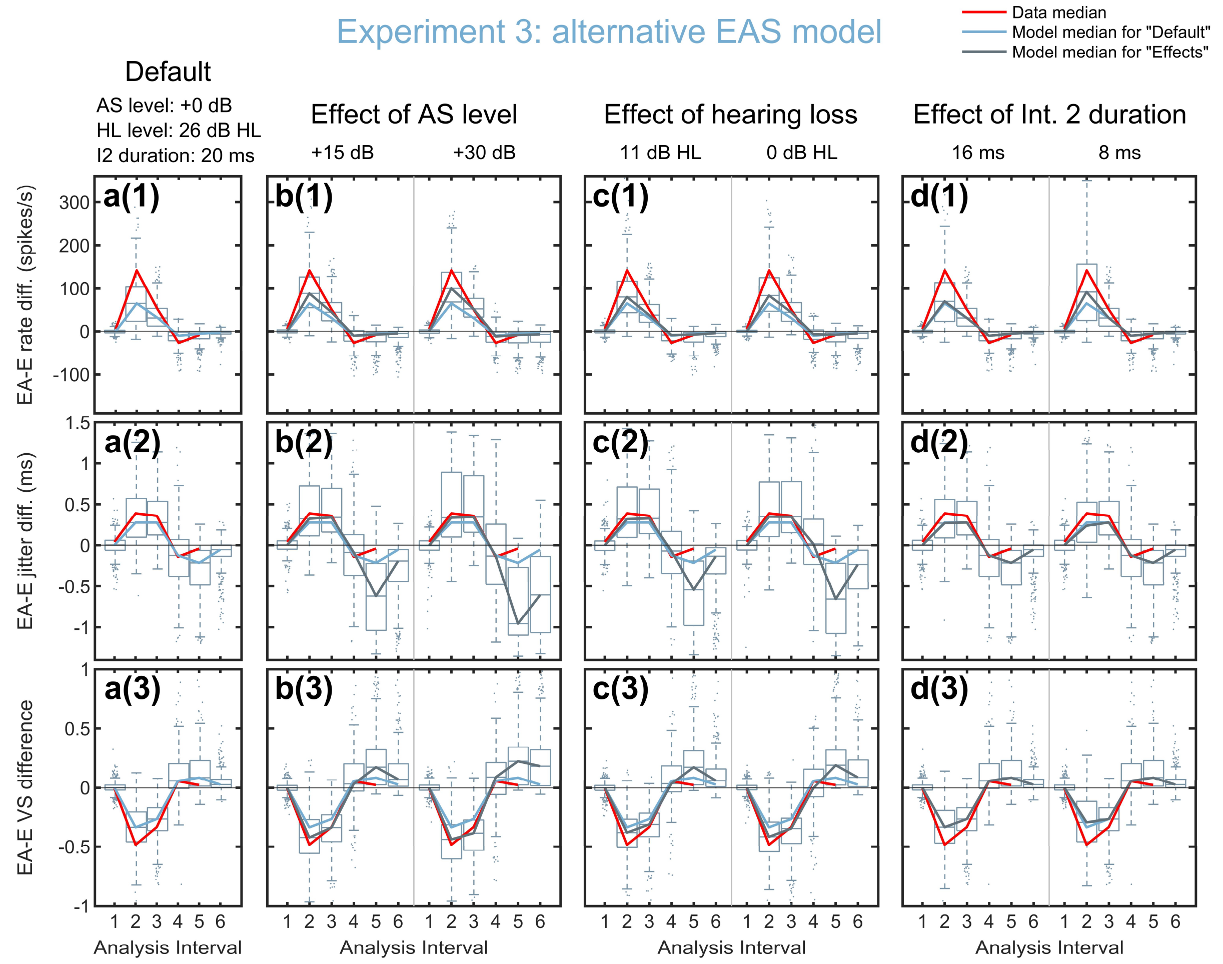

Figure ESM 5: Additional parametric analysis for experiment 3. Corresponds to Figure 11 from the main manuscript.

a Summarized predictions of the alternative EAS model for experiment 3 as shown in figure Figure 5 in the main text. The red line represents the median of the experimental data.
b-d Effect of AS level (**b**), HL level (**c**), and duration of analysis interval I2 (**d**) on the predictions of the alternative EAS model. The medians of the experimental data and the "default" simulation are depicted with red and light blue lines, respectively.

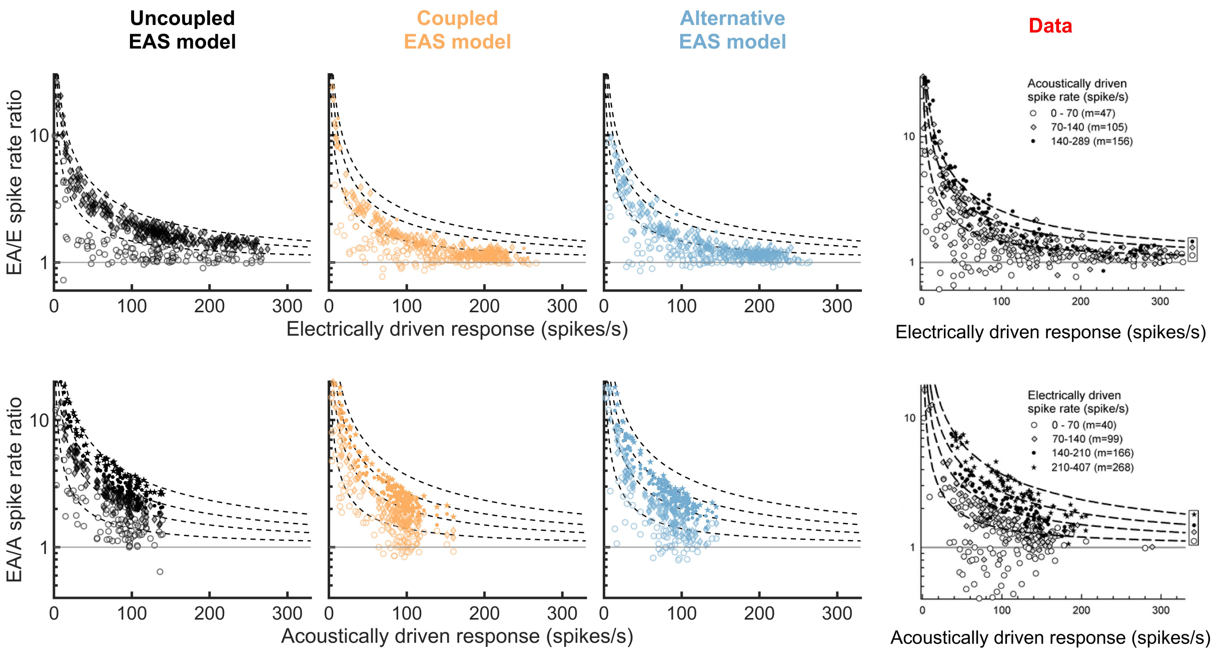

Figure ESM 6: Additional results for experiment 3. Corresponds to Figure 6 from the main manuscript.

Sustained spike rates measured during analysis interval I3. Top row: ratios of spike rates evoked by the electric+acoustic stimuli (EA) and the electric-only stimulus (E), plotted against the electric-only rate. Bottom row: same as in the top panels, but using the acoustically driven spike rate (A) instead of the electrically driven rate. Predictions of the different EAS model variants are compared to experimental data of Miller et al. (2009).

The ANFs have been grouped according to their acoustically driven (top row) or electrically driven (bottom row) spike rate as indicated in the legends. The dashed lines indicate the theoretical curve for each group median m of the experimental ANF population, assuming linear addition of E+A spike rates. They are identical across the panels in each row, allowing for a better orientation when comparing simulated and experimental results.

The data have been reprinted by permission from the Association for Research in Otolaryngology: Springer Nature, JARO - Journal of the Association for Research in Otolaryngology, Miller et al. (2009), Copyright (2009)
